# Dynamic Interfacial pH Stabilization and (002) Oriented Deposition Enabled by Histidine‐Induced Solid Electrolyte Interphase for Highly Reversible Zn Anodes

**DOI:** 10.1002/advs.76765

**Published:** 2026-07-31

**Authors:** Qi Liu, Yimin Chen, Jianwei Lu, Xiangqun Zhuge, Xiyuan Zhong, Huaichong Sun, Yibing Li, Zhihong Luo, Kun Luo, Weiwei Lei, Dan Liu, Anjun Hu, Aijing Ma

**Affiliations:** ^1^ Guangxi Key Laboratory of Optical and Electronic Materials and Devices, Collaborative Innovation Center For Exploration of Nonferrous Metal Deposits and Efficient Utilization of Resources in Guangxi, College of Materials Science and Engineering Guilin University of Technology Guilin Guangxi China; ^2^ Jiangsu Province Engineering Research Center of Intelligent Manufacturing Technology for the New Energy Vehicle Power Battery, School of Materials and Engineering Changzhou University Changzhou China; ^3^ School of Science, STEM College RMIT University Melbourne Victoria Australia; ^4^ College of Materials and Chemistry & Chemical Engineering (College of Lithium Resources and Lithium Battery Industry) Chengdu University of Technology Chengdu China; ^5^ State Key Laboratory of Advanced Separation Membrane Materials, School of Chemical Engineering and Technology Tiangong University Tianjin China

**Keywords:** (002) facet, aqueous zinc ion batteries, interfacial pH, oriented plating/stripping, SEI

## Abstract

Aqueous zinc batteries hold great promise for large‐scale energy storage due to their high energy density, safety, and cost‐effectiveness. However, the intrinsic thermodynamic instability of zinc drives inevitable HER, leading to interfacial accumulation of OH^−^ that significantly exacerbates dendrite growth and “dead zinc” formation. This work leverages the specific structural and reactive properties of histidine (HIS) to construct a Zn(OH)_2_‐HIS ultrathin solid electrolyte interphase (SEI) on the zinc anode. This SEI stabilizes the interfacial pH via a synergistic mechanism of chemical buffering and physical blocking. Chemically, the imidazole and amino groups buffer the pH via reversible protonation/deprotonation; physically, the SEI disrupts the interfacial hydrogen‐bond network and repels solvated water, thereby suppressing H_2_O‐induced side reactions. Additionally, the SEI modulates interfacial surface energy to enable (002)‐oriented deposition. Consequently, the HIS@Zn anode achieves significantly improved reversibility with a high Coulombic efficiency of 99%. It exhibits ultra‐stable cycling for over 1350 h at 10 mA cm^−2^ and 5 mAh cm^−2^. Even at a high depth of discharge of 81%, stable operation is maintained for over 300 h. Furthermore, the HIS@Zn||MnO_2_ full cell delivers an initial capacity of 146.6 mAh g^−1^ at 1 A g^−1^, retaining 92.33% of its capacity after 700 cycles.

## Graphical abstract

The histidine‐induced ultrathin SEI (Zn(OH)_2_‐HIS) delivers exceptional anode stability by stabilizing the interfacial pH via a synergistic mechanism of chemical buffering and physical blocking, while inducing dendrite‐free oriented (002) deposition.

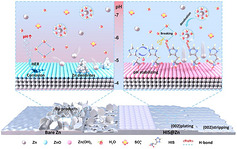

## 1 Introduction

In recent years, aqueous zinc‐ion batteries (AZIBs) have garnered increasing attention owing to their high theoretical capacity (820 mAh g^−1^), low redox potential (−0.76 V vs. SHE), intrinsic safety, and environmental friendliness [1, 2]. Nevertheless, critical challenges remain, particularly severe dendrite growth and parasitic reactions, which substantially hinder the practical application of zinc metal anodes and the large‐scale commercialization of AZIBs. To mitigate the performance degradation of zinc anodes, extensive efforts have been devoted to electrolyte and interfacial engineering, highlighting the crucial roles of the electrical double layer (EDL) structure, ion diffusion behavior, and solvation configuration in governing anode stability [3]. Despite these advances, the dynamic regulation of interfacial pH and its specific impact on zinc stripping behavior have not yet been systematically investigated.

According to the Pourbaix diagram, the standard electrode potential of the hydrogen evolution reaction (HER, 0 V vs. SHE) is significantly higher than that of the Zn^2+^/Zn couple (−0.76 V vs. SHE), rendering HER thermodynamically inevitable during plating. Recent studies have further demonstrated that freshly exposed zinc surfaces during the stripping process are also highly susceptible to HER [4]. The continuous consumption of H^+^ severely perturbs the local interfacial environment, leading to a sharp increase in OH^−^ concentration and subsequently inducing the formation of by‐products (e.g., Zn_4_SO_4_(OH)_4_·xH_2_O). This process not only results in irreversible Zn^2+^ loss but also causes a pronounced increase in interfacial resistance and surface deterioration. Therefore, developing effective interfacial pH stabilization strategies is of great importance for suppressing HER, mitigating parasitic reactions, and enhancing the reversibility of zinc metal anodes.

To address the inevitable pH fluctuations in aqueous electrolytes, various buffering additives have been explored. For instance, weak acid ammonium salts [5] such as CH_3_COONH_4_ [6] and NH_4_H_2_PO_4_ [7] can regulate interfacial pH by capturing OH^−^/H^+^ through NH_4_^+^ and weak acid anions. Similarly, nitrogen‐containing heterocycles (e.g., pyridine and imidazole) [8, 9] and amino acids featuring zwitterionic groups (‐COO^−^/‐NH_3_^+^) [10, 11] are capable of stabilizing pH through reversible proton transfer processes. However, these additives often suffer from limited Zn^2+^ transport kinetics due to competitive cation migration and increased electrolyte viscosity, while their buffering efficacy is intrinsically restricted by bulk diffusion limits. More recently, sacrificial buffering additives have been proposed to decompose at the Zn anode, forming an in situ form solid electrolyte interphase (SEI) that enables concurrent pH regulation and stability enhancement. For example, Li et al. [8]. reported that valerolactam (VL) undergoes ring‐opening polymerization to form a mechanically robust SEI. Ouyang et al. [12]. demonstrated that taurine provides effective pH regulation via the synergistic buffering action of its amine and sulfonic acid groups; simultaneously, the as‐ formed organic/inorganic hybrid SEI substantially enhance resistance to parasitic side reactions. Nevertheless, sacrificial additives are significantly affected by the local chemical environment during SEI conversion, often leading to compositional and structural inhomogeneities. Furthermore, most existing studies primarily focus on the plating process, paying insufficient attention to the stripping process and the dynamic interfacial environment, thereby limiting a comprehensive understanding of zinc anode reversibility.

**SCHEME 1:**
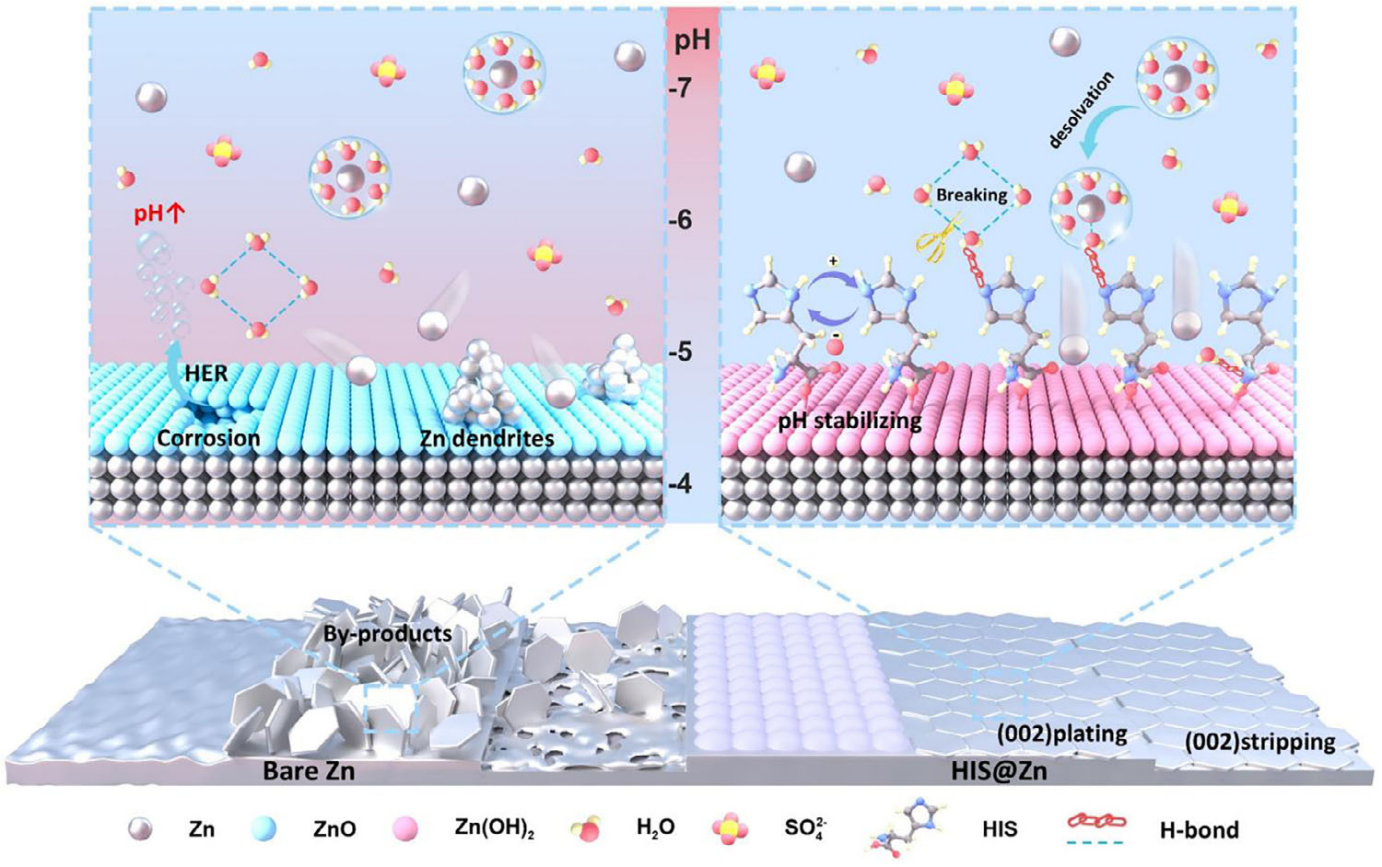
Schematic illustrations of the surface morphologies and Zn plating/stripping processes on bare Zn and HIS@Zn electrodes, with enlarged views of the corresponding electrode/electrolyte interfacial microenvironments.

Herein, we construct an ultrathin Zn(OH)_2_ and HIS coordinated SEI (Zn(OH)_2_‐HIS) on the zinc anode (denoted as HIS@Zn) by leveraging the intrinsic imidazole, amino, and carboxyl functional groups of histidine, as well as strong coordination affinity with Zn^2+^. This interfacial layer effectively addresses the uncontrolled pH rise, vigorous HER, byproduct accumulation, and dendritic growth encountered by pristine zinc anodes (Scheme 1). Benefiting from the reversible protonation/deprotonation of imidazole (N^τ^) and amino groups (‐NH_2_), HIS@Zn captures H^+^ and neutralizes OH^−^ to buffer drastic interfacial pH fluctuations. Meanwhile, it disrupts the interfacial hydrogen bond network and displaces solvated water molecules, thereby suppressing H_2_O‐related side reactions and enhancing interfacial Zn^2+^ transport. Furthermore, the Zn(OH)_2_‐HIS SEI regulates the energy barriers for zinc deposition and dissolution, promoting Zn(002) oriented plating/stripping. As a result, HIS@Zn||HIS@Zn symmetric cells exhibit stable cycling for over 1350 h at 10 mA cm^−2^ with an areal capacity of 5 mAh cm^−2^. Even under a high areal capacity of 20 mAh cm^−2^ and a deep discharge depth of 81%, stable operation exceeding 300 h is achieved. Furthermore, HIS@Zn||MnO_2_ full cells deliver an initial capacity of 146.6 mAh g^−2^ at 1 A g^−1^ and retain 92.33% of the capacity after 700 cycles, demonstrating outstanding long‐term cycling stability and practical potential.

## 2 Result and Discussion

### 2.1 Structural Characteristics and Stability of the SEI

**FIGURE 1:**
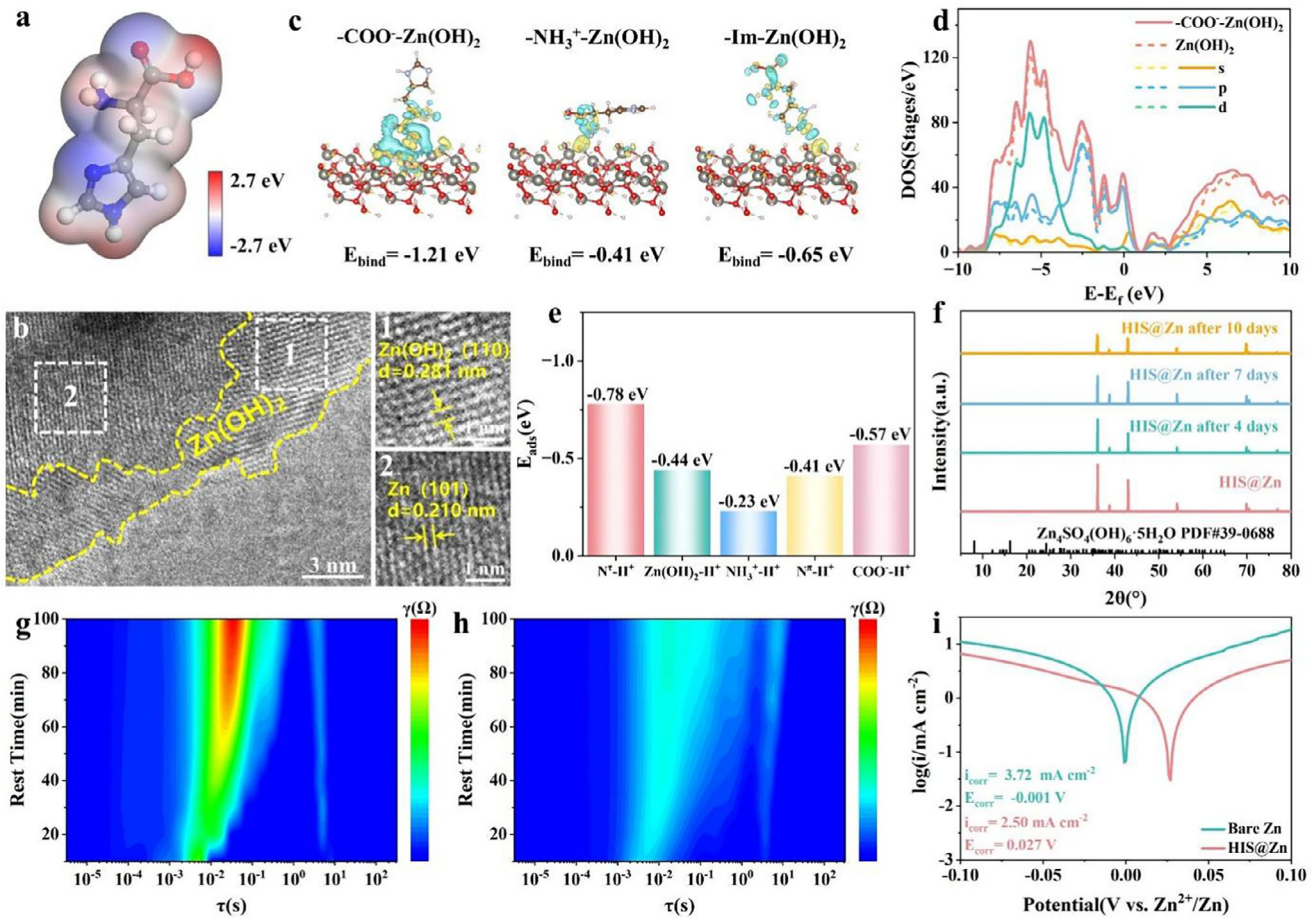
(a) ESP of the HIS molecule. (b) HRTEM of the material scraped from the HIS@Zn. (c) Binding energies between different functional groups and Zn(OH)_2_. (d) Comparison of density of states (DOS) between Zn(OH)_2_ and ‐COO^−^‐Zn(OH)_2_. (e) H^+^ adsorption energies at the different sites. (f) XRD patterns of HIS@Zn during the immersion process. (g, h) DRT analysis of Zn||Zn symmetric cells by operando EIS evaluation during resting. (i) Tafel curves and corresponding corrosion currents and potentials.

Figure 1a shows the electrostatic potential distribution of histidine (HIS), compared with water molecules (Figure S1), HIS exhibits a markedly heterogeneous charge distribution. Specifically, the electron cloud is enriched around the non‐protonated N^τ^ center of the imidazole group (‐Im), forming a negatively charged region, which is relatively low near the hydrogen atoms at the ‐COOH terminal, giving a positive electrostatic potential. Moreover, the dipole moment of HIS is significantly larger than that of H_2_O (Figure S2), indicating a stronger propensity for interfacial interaction with zinc. Given that the isoelectric point of histidine (pl ≈ 7.59) lies in the weakly alkaline regime, dissolution of HIS in water spontaneously drives the acid–base equilibrium toward this pH value, rendering the solution mildly alkaline (Figure S3) [13]. Under such conditions, OH^−^ can readily react with amphoteric Zn, promoting the formation of hydroxide species. Consistently, the solution pH decreases from 7.61 to 7.49 after zinc foil immersion (Figure S4a,b), indicating the consumption of OH^−^ during interfacial reactions. SEM images (Figure S5a,b) further reveal that HIS@Zn exhibits a uniform micro‐topographical morphology, confirming that chemical reactions occur between the zinc electrode and HIS solution, accompanied by surface reconstruction.

High‐resolution transmission electron microscopy (HRTEM) and selected‐area electron diffraction (SAED) analyses demonstrate the presence of a Zn(OH)_2_ layer with an average thickness of approximately 4 nm within the SEI. Diffraction signals corresponding to the (110), (006), (300), and (119) planes of Zn(OH)_2_ are clearly detected (Figure 1b and Figure S6), verifying the formation of an inorganic Zn(OH)_2_ phase in the SEI. FTIR spectroscopy was recorded and is shown in Figure S7, the O‐H stretching vibration at 3223 cm^−1^ and the Zn‐O vibration at 528 cm^−1^ in HIS@Zn confirm the existence of Zn(OH)_2_ [14]. Meanwhile, the asymmetric and symmetric stretching vibrations of carboxyl shift from 1618 and 1456 cm^−1^ to 1616 and 1415 cm^−1^, respectively, indicating coordination interactions between carboxyl groups and Zn(OH)_2_. Density functional theory (DFT) calculations further reveal (Figure 1c) that carboxyl exhibits the highest binding energy with Zn(OH)_2_ (−1.21 eV) and the most pronounced charge transfer behavior. Density of states (DOS) analysis (Figure 1d and Figure S8) shows a significant enhancement of electronic states in the −8 to −2 eV energy range upon carboxyl coordination, suggesting substantial electronic structure reconstruction. In this region, a strong overlap between O2p and Zn3d orbitals is observed, evidencing robust coordination between the carboxyl oxygen and Zn centers in Zn(OH)_2_. In contrast, ‐Im and ‐NH_3_^+^ display much lower binding energies and weaker orbital coupling with Zn(OH)_2_, indicating relatively weak interactions. These results collectively confirm the formation of a Zn(OH)_2_‐HIS coordinated hybrid SEI via Zn‐O bond.

Differential capacitance obtained by AC voltammetry (Figure S9) shows that HIS@Zn exhibits a substantially reduced capacitance compared with bare Zn, further confirming SEI formation at the interface. Contact angle measurements (Figure S10) indicate that HIS@Zn displays a smaller contact angle (69.5°) than bare Zn (84.6°), demonstrating that the organic/inorganic hybrid SEI enhances electrolyte wettability. To evaluate the stability of SEI, HIS@Zn was immersed in 2 M ZnSO_4_ electrolyte for 24 h, after which a continuous Zn(OH)_2_ layer with an average thickness of ≈ 4 nm remained intact (Figure S11). Even after immersion for over 10 days, the surface remains stable without byproducts (Figure 1f and Figure S12b). In contrast, bare Zn exhibits characteristic diffraction peaks of Zn_4_SO_4_(OH)_6_·5H_2_O (ZSH) after only 4 days of immersion (Figure S13), with peak intensities increasing over time. SEM images after 10 days clearly reveal randomly oriented hexagonal platelet‐like ZSH deposits on the Zn surface (Figure S12a). Furthermore, DFT calculations indicate that the H^+^ adsorption energy of N^τ^, NH_3_^+^, N^π^, and COO^−^ sites is −0.78, −0.23, −0.41, and −0.57 eV, respectively. Typically, the adsorption energy of N^τ^ is more negative than Zn(OH)_2_ (−0.44 eV), demonstrating a stronger affinity for H^+^ (Figure 1e and Figure S14). This preferential H^+^ capture effectively protects the inner inorganic Zn(OH)_2_ layer, endowing the SEI with enhanced interfacial stability.

EIS plots during the resting process (Figure S15a,b) reveal that the charge‐transfer resistance (*R_ct_*) of bare Zn cells increases markedly with resting time, whereas HIS@Zn exhibits only a slight resistance increase from 10 to 100 min and maintains a much lower *R_ct_* value compared to bare Zn. To further elucidate the electrochemical processes, the distribution of relaxation times (DRT) analysis was employed to investigate the modified electrodes. Two dominant relaxation processes during resting are identified: interfacial Zn^2+^ charge transfer (τ≈ 10^−2^ s) and Zn^2+^ diffusion (τ≈10^0^ – 10^1^ s) [15]. As shown in Figure 1g,h, the relaxation peak intensity of bare Zn at τ≈ 10^−2^ s increases continuously with resting time, indicating progressive accumulation of ZSH by‐products at the interface. In contrast, HIS@Zn exhibits minimal peak variation with stable intensity, demonstrating effective suppression of interfacial byproduct formation by the SEI. Tafel curves further reveal (Figure 1i) that HIS@Zn exhibits a more positive corrosion potential (*E_corr_* = 0.027 mV) and a lower corrosion current density (*i_corr_* = 2.50 mA cm^−2^) than bare Zn (*E_corr_* = −0.001 mV; *i_corr_* = 3.72 mA cm^−2^), confirming the superior corrosion resistance and interfacial stability afforded by the HIS‐derived SEI.

### 2.2 pH Buffering Mechanism at the Electrode/Electrolyte Interface

**FIGURE 2:**
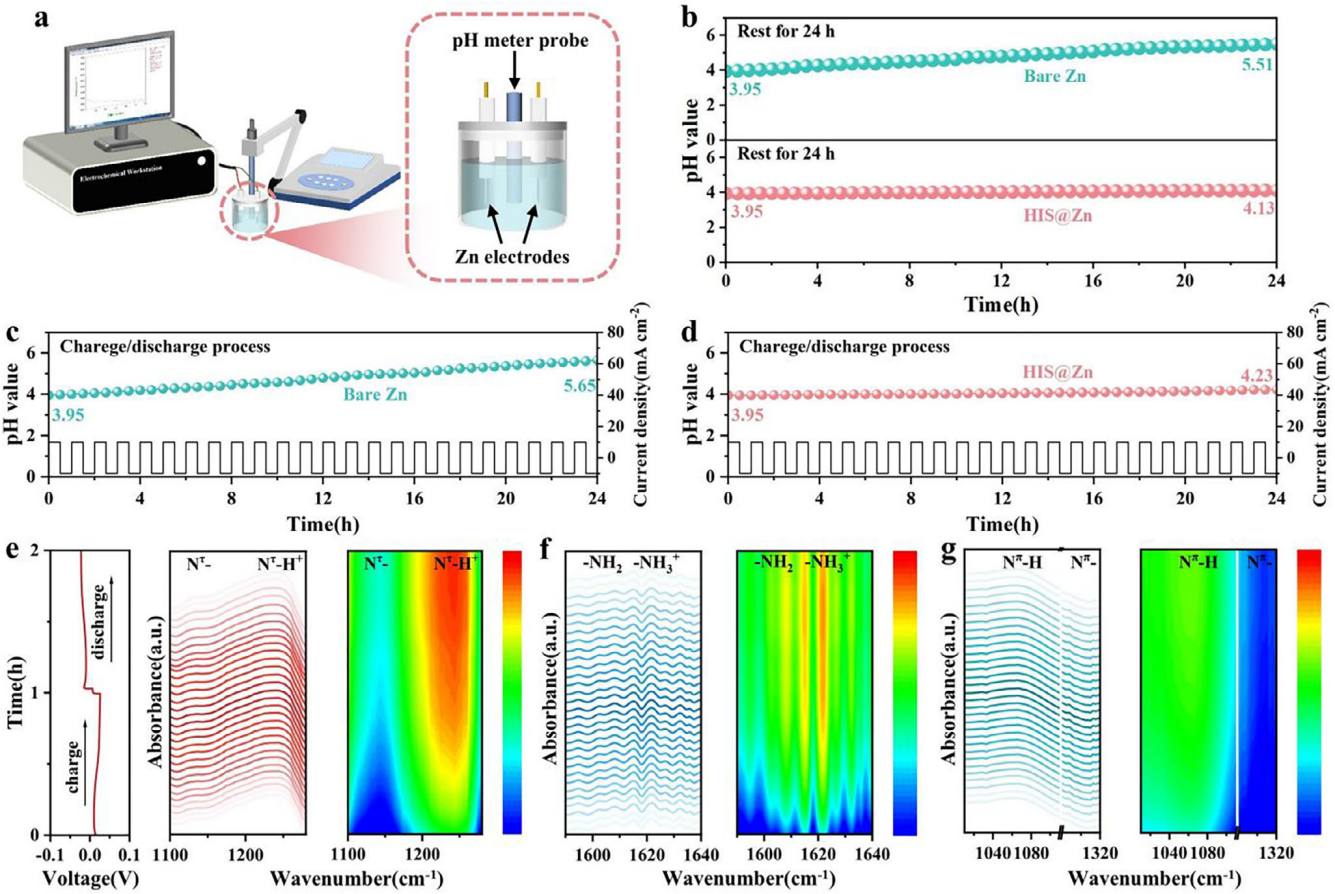
(a) Schematic diagram of the home‐made in situ interfacial pH testing equipment. (b) In situ pH test of Zn||Zn cells with bare Zn and HIS@Zn during the rest process and (c, d) during the charge/discharge process. (e, f, g,) In situ FTIR spectra and corresponding contour maps of the Zn||Zn cell with HIS@Zn during the charge/discharge process.

A home‐made in situ pH monitoring system (Figure 2a and Figure S16) was employed to continuously track the pH evolution at the electrode/electrolyte interface of zinc electrodes during both resting and electrochemical cycling. As shown in Figure 2b, during the resting period, the interfacial pH adjacent to bare Zn rapidly increases from an initial value of 3.95 to 5.51. In sharp contrast, the interfacial pH of the HIS@Zn electrode exhibits only a slight increase, reaching merely 4.13. A similarly pronounced discrepancy is observed during the electrochemical charge/discharge process. As depicted in Figure 2c,d, after cycling at 10 mA cm^−2^ and 5 mAh cm^−2^ for 24 h, the interfacial pH of bare Zn rises steeply from 3.95 to 5.65, whereas that of HIS@Zn increases only marginally to 4.23 under the same conditions. Even though the current density and capacity are as high as 16 mA cm^−2^ and 16 mAh cm^−2^, respectively, the interfacial pH of HIS@Zn only rises to 4.31, which reaches 5.84 for bare Zn (Figure S17 and S18), demonstrating the effective pH buffering of HIS@Zn under harsh conditions. These results clearly indicate that persistent HER occurs at the bare Zn interface during both resting and cycling, leading to substantial local OH^−^ accumulation, which in turn induces the formation of ZSH byproducts and promotes non‐uniform zinc plating/stripping. By contrast, the SEI formed on HIS@Zn effectively suppresses the rapid interfacial pH rise, thereby enhancing interfacial stability and inhibiting by‐product formation and dendrite growth.

The observed pH stabilization is closely associated with the reversible protonation /deprotonation behavior of the ‐Im and ‐NH_2_ groups in HIS. In situ FTIR spectroscopy was conducted to further elucidate the underlying mechanism. The characteristic vibrational modes of N^τ^‐ and N^τ^‐H^+^ in the imidazole ring appear at approximately 1105 and 1265 cm^−1^, respectively, while those of N^π^‐ and N^π^‐H are located near 1088 and 1286 cm^−1^. In addition, the vibrations of ‐NH_2_ and ‐NH_3_^+^ are observed at around 1602 and 1620 cm^−1^, respectively [16]. As shown in Figure 2e–g, the intensity of the N^τ^‐H^+^ vibrational peak progressively increases and remains at a consistently high‐level during cycling. This behavior is attributed to the rapid migration of H^+^ toward the electrode/electrolyte interface at the initial stage of charging, where N^τ^‐ sites promptly capture protons to form N^τ^‐H^+^, resulting in the enhanced peak intensity. Meanwhile, the probable OH^−^ generated at the interface is rapidly neutralized by N^τ^‐H^+^, and the regenerated N^τ^‐ immediately binds with H^+^ to form N^τ^‐H^+^ (reactions 1 and 2). Owing to the fast protonation/deprotonation kinetics between N^τ^‐ and N^τ^‐H^+^, the corresponding vibrational intensity remains persistently high during zinc plating/stripping, effectively preventing H^+^‐induced zinc corrosion and responding rapidly to OH^−^ generation simultaneously, thereby suppressing by‐product formation. A similar buffering behavior is observed for the ‐NH_2_/‐NH_3_^+^ pair (reactions 3 and 4), albeit with relatively weaker peak intensities. In contrast, the intensity of N^π^‐H remains essentially unchanged throughout cycling, and no N^π^‐ vibration is detected. These results unambiguously demonstrate that the N^τ^‐ site of the imidazole ring and the ‐NH_2_ group serve as the dominant pH‐buffering centers, with the N^τ^‐ site playing a primary role in interfacial pH regulation.

(1) $$-N^{\tau }+H^{+}\to -N^{\tau }-H^{+}$$

(2) $$-N^{\tau }-H^{+}+OH^{-}\to -N^{\tau }+H_{2}O$$

(3) $$-NH_{2}+H^{+}\to -N{H_{3}}^{+}$$

(4)

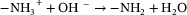




### 2.3 Regulation of Interfacial Hydrogen Bond Network and Solvation Structure

**FIGURE 3:**
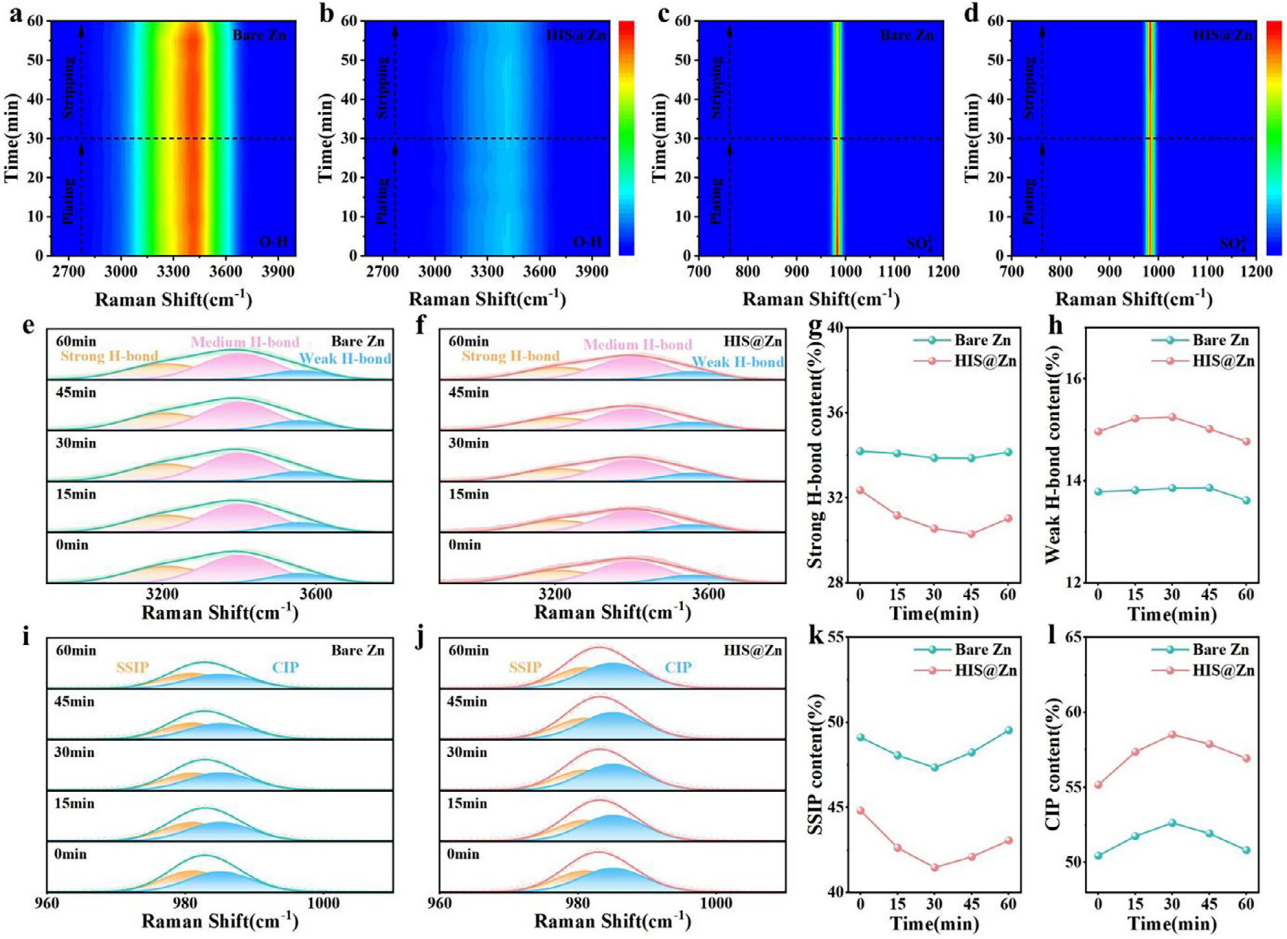
In situ Raman spectra of the electrode/electrolyte interface in bare Zn and HIS@Zn anodes, showing (a, b) the O‐H stretching region and (c, d) SO_4_^2−^ stretching region. (e, f) Raman spectra of the O─H bond and (g, h) corresponding ratio of Strong H─bond and Weak H─bond. (i, j) Raman spectra of SO_4_2^−^ signals during different plating and stripping times and (k, l) corresponding ratio of SSIP, CIP.

Interfacial pH stabilization may also originate from the suppression of H_2_O‐related parasitic reactions. In situ Raman spectroscopy was employed to further elucidate the interfacial dynamic evolution during plating/stripping. As shown in Figure 3a,b, the bare Zn electrode exhibits a pronounced increase in the O‐H stretching signal (3000 – 3800 cm^−1^) during cycling, indicating continuous accumulation of free water molecules within the electrical double layer (EDL). In contrast, the O‐H signal intensity variation at the HIS@Zn interface is significantly attenuated, suggesting a markedly lower interfacial water concentration. This result demonstrates that the SEI may promote the formation of a water‐deficient environment at the interface. Meanwhile, a rapid decay of the SO_4_^2−^ Raman signal (981 – 985 cm^−1^) is observed at the bare Zn interface (Figure 3c), which can be attributed to parasitic reactions (Zn^2+^ + OH^−^ + ZnSO_4_ + H_2_O → Zn_4_SO_4_(OH)_6_·5H_2_O↓) that continuously consume both SO_4_^2−^ and Zn^2+^. In sharp contrast, the SO_4_^2−^ signal intensity at the HIS@Zn interface remains stable throughout cycling (Figure 3d). The evolution of the SO_4_^2−^ signal also reflects the dynamic distribution of Zn^2+^ at the electrode/electrolyte interface. At the bare Zn interface, sustained Zn^2+^ consumption further induces concentration polarization, exacerbating non‐uniform nucleation and dendrite growth. Conversely, the HIS@Zn interface maintains a more homogeneous and continuous Zn^2+^ flux, facilitating uniform zinc plating and stripping.

The influence of the SEI on the interfacial hydrogen bond network and solvation structure was further analyzed. As shown in Figure 3e,f, the O‐H stretching vibrations can be deconvoluted into three components corresponding to a strong hydrogen bond (3209 cm^−1^), a moderate hydrogen bond (3400 cm^−1^), and a weak hydrogen bond (3556 cm^−1^). During cycling, both bare Zn and HIS@Zn systems exhibit similar trends in the evolution of strong and weak hydrogen bonds (Figure 3g,h). However, compared with bare Zn, HIS@Zn displays a lower proportion of strong hydrogen bonds and a higher proportion of weak hydrogen bonds, possibly due to the donors/acceptors of hydrogen bonds within the SEI forming hydrogen bonds with water molecules, thereby disrupting the original hydrogen bond network among water molecules and reducing water activity. The SO_4_^2−^ spectral region can be further resolved into two dominant ion‐pairing configurations (Figure 3i,j): solvent‐separated ion pairs (SSIP, [Zn^2+^(H_2_O)_6_·SO_4_^2−^] at 981 cm^−1^) and contact ion pairs (CIP, [Zn^2+^(H_2_O)_5_·SO_4_^2−^] at 985 cm^−1^) [17]. Both systems exhibit dynamic evolution from SSIP to CIP during cycling (Figure 3k,l), however, the HIS@Zn interface shows a markedly reduced SSIP fraction and a significantly increased CIP fraction, indicating that donors/acceptors of hydrogen bonds within the SEI interact with solvated water molecules and reduce the number of coordinated water molecules in solvated Zn^2+^ ions. The disruption of the hydrogen‐bond network and the reduction in solvated water synergistically suppress H_2_O‐related parasitic reactions [18]. LSV curves further reveal that HIS@Zn exhibits a more negative overpotential accompanied by lower current density for HER than bare Zn (Figure S19), confirming the effective inhibition of HER by the SEI. In addition, Arrhenius analysis (Figure S20) yields a lower activation energy (*E_a_* = 34.92 kJ mol^−1^) for HIS@Zn compared with bare Zn (*E_a_* = 42.52 kJ mol^−1^), demonstrating that the SEI substantially reduces the Zn^2+^ desolvation energy barrier.

According to the above‐mentioned results, the SEI stabilizes the electrode/electrolyte interfacial environment via a synergistic “chemical buffering‐physical blocking” mechanism, thereby enabling long‐term interfacial pH stability. On the one hand, the N^τ^ and ‐NH_2_ sites within the SEI dynamically capture H^+^ and neutralize accumulated OH^−^ through reversible protonation/deprotonation, chemically suppressing HER‐induced local alkalization. On the other hand, the SEI destroy the interfacial hydrogen bond network and interacts with solvated water, significantly reducing the free water content in the EDL and solvation structure, which physically mitigates H_2_O‐related parasitic reactions. These synergistic effects not only stabilize the interfacial chemistry but also create favorable conditions for uniform zinc plating/stripping.

### 2.4 Zn^2+^ Transport Kinetics and Deposition Behavior

**FIGURE 4:**
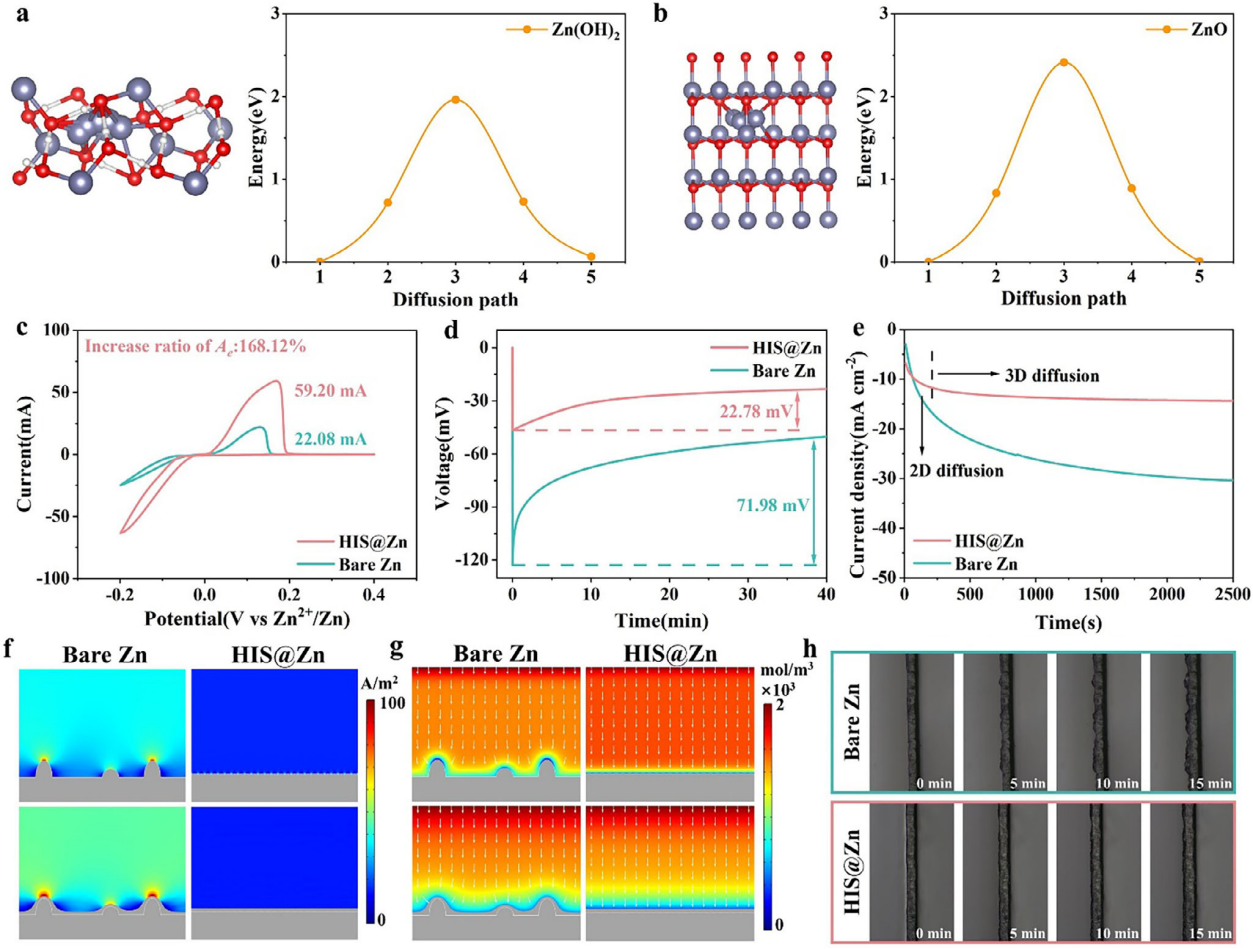
(a, b) Molecular models and migration energy barrier of Zn^2+^ in Zn(OH)_2_ and ZnO. (c) CV curves of Zn||SS cells with bare Zn and HIS@Zn. (d) Nucleation overpotential of Zn||Zn cells with bare Zn and HIS@Zn. (e) Chronoamperometry curves of Zn||Zn cells with bare Zn and HIS@Zn at a constant overpotential of −0.15 V. COMSOL simulation of (f) the current field and (g) Zn^2+^ concentration field distributions of bare Zn and HIS@Zn during the plating process. (h) In situ optical microscopy observations in the bare Zn and HIS@Zn anodes at the current density of 10 mA cm^−2^.

The transport behavior of Zn^2+^ plays a crucial role in determining the uniformity of zinc plating/stripping. DFT calculations were performed to evaluate the Zn^2+^ diffusion energy barriers on the inorganic components ZnO and Zn(OH)_2_ (Figure 4a,b). ZnO exhibits a high diffusion barrier of 2.41 eV, whereas Zn(OH)_2_ presents a substantially lower barrier of 1.96 eV, indicating more favorable Zn^2+^ migration kinetics on Zn(OH)_2_. The Zn^2+^ transference number was further evaluated using the Bruce‐Vincent method, revealing a remarkable increase from 0.21 for bare Zn to 0.76 for HIS@Zn (Figure S21a,b). Moreover, Warburg fitting of the low‐frequency region of EIS spectra shows that the Zn^2+^ diffusion coefficient of HIS@Zn reaches 3.25×10^−13^ cm^2^ s^−1^, which is nearly two orders of magnitude higher than that of bare Zn (8.45×10^−15^ cm^2^ s^−1^), as shown in Figure S22a,b. These results collectively demonstrate that HIS@Zn possesses significantly accelerated Zn^2+^ transport kinetics and enhanced directional Zn^2+^ migration capability. CV plots in Figure 4c show that the peak current of HIS@Zn||SS (59.20 mA) is markedly higher than that of Zn||SS (22.08 mA). Correspondingly, the electrochemically active surface area (*A_e_*) of HIS@Zn is increased by 168.12% relative to bare Zn, indicating that the unique micro‐topographical structure of HIS@Zn provides a greater number of active nucleation sites for Zn^2+^. Nucleation overpotential analysis (Figure 4d) further reveals a much lower nucleation overpotential of 22.78 mV for HIS@Zn compared with 71.98 mV for bare Zn. The reduced nucleation overpotential suggests that HIS@Zn effectively lowers the Zn^2+^ nucleation energy barrier, thereby promoting uniform nucleation. CA curves (Figure 4e) show that Zn^2+^ deposition on HIS@Zn rapidly transitions from initial 2D diffusion to stable 3D diffusion, leading to uniform planar deposition. In contrast, bare Zn remains dominated by 2D diffusion for a prolonged period, with the current density continuously increasing even after 2500 s, indicative of dendrite‐prone growth behavior.

COMSOL Multiphysics simulations further corroborate these findings (Figure 4f,g). For bare Zn, surface defects induce localized enrichment of electric field intensity and ion concentration, which enhances the attraction of Zn^2+^ toward tip regions and promotes preferential deposition at these sites, ultimately resulting in severe dendrite formation. By contrast, the uniform micro‐topography of the HIS@Zn electrode effectively homogenizes the interfacial electric field and concentration distributions, providing robust regulation for uniform zinc deposition and dendrite suppression. As deposition proceeds, a dense and smooth zinc layer is ultimately formed. To directly visualize the dynamic deposition behavior, in situ optical microscopy was conducted at a current density of 10 mA cm^−2^ (Figure 4h). Bare Zn exhibits mossy deposits after only 5 min, which rapidly evolve into irregular and rough deposition layers. In sharp contrast, HIS@Zn consistently displays uniform, smooth, and dendrite‐free deposition morphologies throughout the deposition process, demonstrating the homogeneous Zn^2+^ deposition and dendrite suppression, thereby significantly enhancing interfacial stability.

### 2.5 Stability and Morphology Evolution During Plating/Stripping Process

**FIGURE 5:**
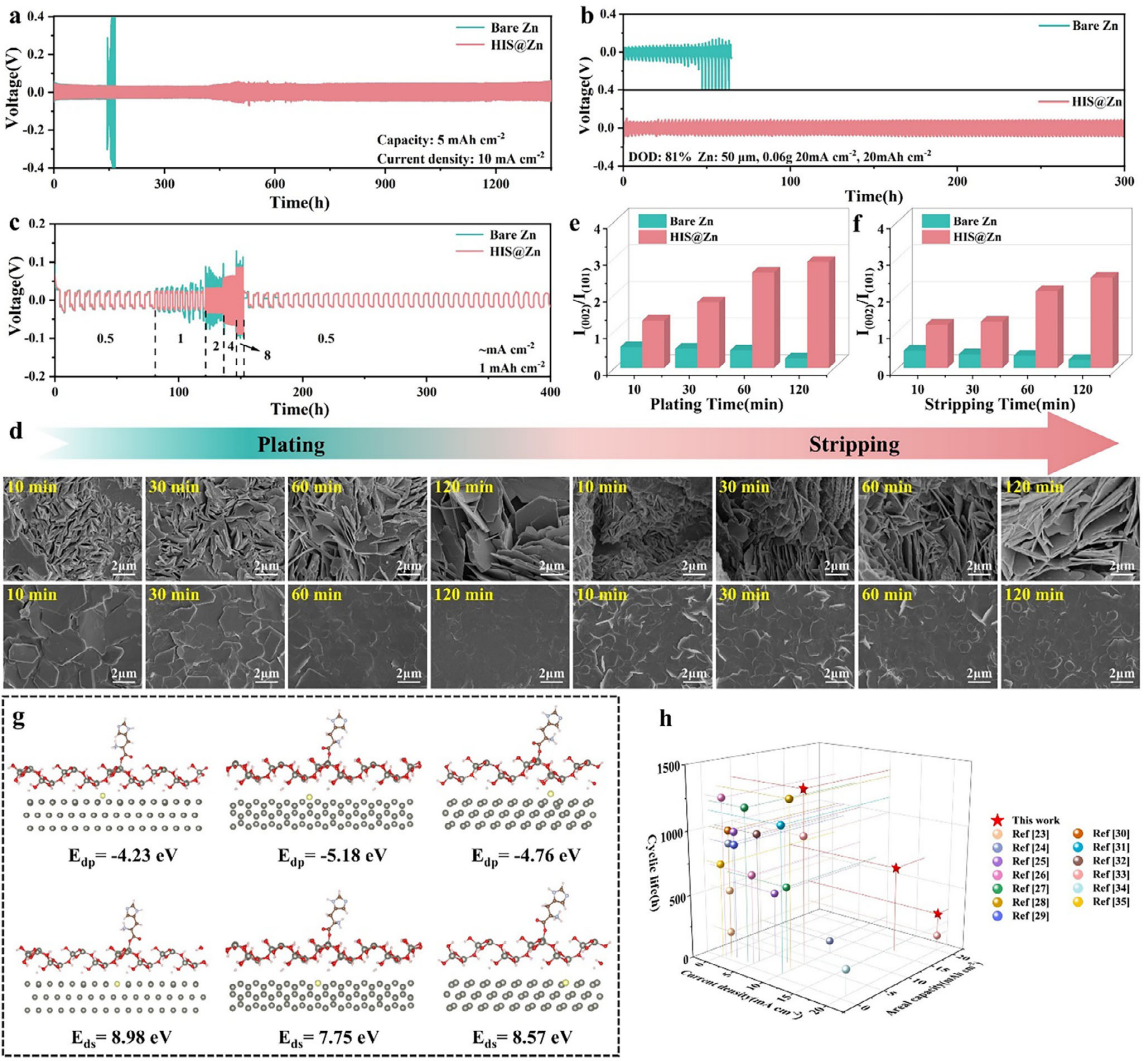
Cyclic performance of Zn||Zn cells with bare Zn and HIS@Zn (a) at the current density of 10 mA cm^−2^ and capacity of 5 mAh cm^−2^, (b) at the current density of 20 mA cm^−2^ and capacity of 20 mAh cm^−2^, (c) rate capability of Zn||Zn cell at a range of 0.5–8 mA cm^−2^. (d) The surface morphology evolution of bare Zn and HIS@Zn during the plating and stripping process at the current density of 10 mA cm^−2^. (e, f) Facet ratio of I_(002)_/I_(101)_ during plating and stripping process. (g) Deposition energy of Zn^2+^ ion on different SEI‐bonded Zn crystal planes and dissolution energy of Zn atom from different SEI‐bonded Zn crystal planes. (h) Comparison of the cycling performance with those reported in recent works.

To evaluate the cycling stability of different electrodes, symmetric cells were assembled using bare Zn, HIS@Zn‐3 h, HIS@Zn and HIS@Zn‐7 h anodes. Under high current densities and large areal capacities of 10 mA cm^−2^ and 5 mAh cm^−2^ (Figure 5a and Figure S23), the bare Zn symmetric cell suffers rapid short‐circuiting after approximately 143 h. Although the HIS@Zn‐3 h and HIS@Zn‐7 h show improved lifespans of 195 h and 446 h, respectively, they still succumb to failure under prolonged cycling. In contrast, the HIS@Zn symmetric cell exhibits a lower initial overpotential and maintains stable cycling for up to 1350 h with a small and steady overpotential, demonstrating a pronounced advantage (Minor voltage fluctuations observed are likely attributable to random measurement errors [14, 19, 20, 21].). Under high depth of discharge (DOD) conditions (Figure S24 and Figure 5b), bare Zn delivers limited cycling lifetimes at both 65% and 81% DOD. By comparison, HIS@Zn sustains stable cycling for ≈500 h at 65% DOD and retains over 300 h of reversible operation even at an extreme DOD of 81%. Rate performance tests further confirm the superior stability of HIS@Zn (Figure 5c). At an areal capacity of 1 mAh cm^−2^, bare Zn exhibits an overpotential increase from 46.8 to 57.3 mV as the current density rises from 0.5 to 1 mA cm^−2^, accompanied by severe voltage fluctuations and eventual short‐circuiting. In contrast, the HIS@Zn symmetric cell displays an initial overpotential of only 39.5 mV at 0.5 mA cm^−2^, which increases moderately to 45.5, 62.6, 112.3, and 159.1 mV at current densities of 1, 2, 4, and 8 mA cm^−2^, respectively. Notably, when the current density is back to 0.5 mA cm^−2^, the overpotential rapidly recovers to 31.3 mV and remains stable for over 400 h, highlighting the excellent robustness of HIS@Zn under dynamically varying current conditions.

As shown in Figure S25a,b, Zn||Cu half‐cell tests further reveal that the initial voltage hysteresis of HIS@Zn is only 50.1 mV, significantly lower than that of bare Zn (67.5 mV). Owing to dendrite formation and parasitic reactions, the Coulombic efficiency of bare Zn fluctuates markedly after ≈100 cycles. In contrast, the HIS@Zn||Cu half‐cell sustains stable cycling for over 3000 cycles with an average Coulombic efficiency of ≈99%, confirming the high reversibility of HIS@Zn.

Subsequently, the differences in plating/stripping behavior between bare Zn and HIS@Zn electrodes were systematically investigated. Morphological evolution under a current density of 10 mA cm^−2^ was monitored. As shown in Figure 5d, bare Zn exhibits a large number of disordered plates with non‐uniform size after only 10 min of deposition. As the deposition time prolonged, these disordered plates gradually grew and stacked, evolving into dendritic structures ultimately. During the stripping process, the appearance of vertically or obliquely Zn plates accompanied by distinct pits is observed as early as 10 min due to the inhomogeneous reaction. Upon extending the stripping time to 30 – 120 min, the plate‐like products further accumulate and enlarge, and the stripping surface becomes increasingly rough, showing a typical characteristic of uneven dissolution behavior. In sharp contrast, HIS@Zn exhibits highly stable interfacial evolution throughout both plating and stripping. At the initial stages, plates with the characteristic of the (002) crystal plane are observed. With the extension of plating/stripping time, the surface becomes progressively denser without noticeable dendrite formation or localized corrosion. These observations are in excellent agreement with the COMSOL simulations and in situ optical microscopy observations.

The evolution of texture during the repeated plating/stripping process was further examined. XRD analysis (Figures S26, S27, and Figure 5e,f) shows that the I_(002)_/I_(101)_ facet ratio of HIS@Zn during plating for 10, 30, 60, and 120 min reaches 1.29, 1.79, 2.60, and 2.89, respectively, which are significantly higher than those of bare Zn (0.57, 0.52, 0.48, and 0.26). During stripping, HIS@Zn consistently maintains high I_(002)_/I_(101)_ values (1.18, 1.26, 2.09, and 2.46), whereas bare Zn remains at much lower levels (0.47, 0.37, 0.33, and 0.22). Notably, no diffraction signals corresponding to ZSH byproducts are detected on HIS@Zn throughout the entire plating/stripping process, while ZSH emerges on bare Zn at the early stage and continuously intensifies with reaction progression.

A similar (002)‐dominated texture phenomenon was observed during the long‐term cycling process. As shown in Figure S28a,b, bare Zn develops extensive loose plate‐like products after 50 and 100 cycles, respectively. Corresponding XRD results confirm a decrease in the I_(002)_/I_(101)_ ratio from 0.26 to 0.20, accompanied by intensified diffraction peaks of byproducts (Figure S29a,b). In contrast, the HIS@Zn symmetric cell maintains a dense and flat plate‐like morphology even after 50 and 1350 cycles (Figure S28c,d). No ZSH characteristic peaks are observed in the XRD patterns, and the I_(002)_/I_(101)_ ratio remains consistently high (Figure S29a,b). These results indicate that the SEI constructed on HIS@Zn effectively suppresses byproduct formation and continuously guides preferential (002)‐oriented plating and stripping.

**FIGURE 6:**
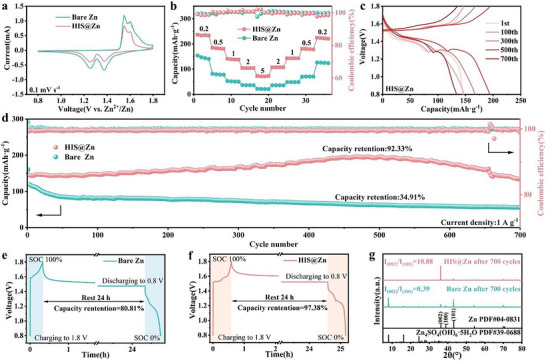
(a) CV curves, (b) Rate performance of Zn||MnO_2_ cells with bare Zn and HIS@Zn. (c) Voltage‐capacity curves of Zn||MnO_2_ cell with HIS@Zn. (d) Cycling performance of Zn||MnO_2_ cells with bare Zn and HIS@Zn at 1 A g^−1^. Self‐discharge curves of Zn|| MnO_2_ cells with (e) bare Zn and (f) HIS@Zn. (g) XRD pattern and corresponding I_(002)_/I_(101)_ facet ratio of HIS@Zn after 700 cycles.

To gain deeper insight into the mechanism of the (002) oriented plating and stripping process, the deposition energies of Zn^2+^ ions and the dissolution energy barriers of Zn atoms on different crystal facets were calculated. As shown in Figure 6g, the Zn^2+^ ion exhibits more negative deposition energies on the Zn(100) and Zn(101) facets than on the Zn(002) facet, indicating a faster deposition rate on Zn(100) and Zn(101). According to Bravais’ law, fast‐growing facets tend to vanish, whereas the slowest‐growing Zn(002) facet becomes the dominant exposed surface [22]. Meanwhile, Zn atoms possess a higher dissolution energy barrier on the Zn(002) facet, leading to preferential dissolution from Zn(100) and Zn(101) during stripping and further enriching the exposure of Zn(002). These results collectively demonstrate that the HIS‐derived SEI effectively regulates the interfacial plating/stripping behavior, inducing preferential Zn(002) orientation, suppressing parasitic reactions and dendrite growth, and ultimately ensuring long‐term stability of the Zn anode. Compared with the reported reference (Figure 5h and Table S1), the HIS@Zn exhibits outstanding cycling performance among the many excellent published articles, underscoring the strong potential of this interfacial engineering strategy for highly reliable aqueous zinc‐ion batteries [23, 24, 25, 26, 27, 28, 29, 30, 31, 32, 33, 34, 35].

### 2.6 Electrochemical Performance of Full Cells

To further evaluate the applicability of the HIS@Zn electrode in enhancing the electrochemical performance of full cells, α‐MnO_2_ cathode materials were synthesized via a hydrothermal method (Figure S30a,b), and HIS@Zn||MnO_2_ full cells were assembled for systematic electrochemical investigations. As shown in the CV curves (Figure 6a), both Zn||MnO_2_ and HIS@Zn||MnO_2_ full cells exhibit two pairs of similar redox peaks, corresponding to the reversible oxidation/reduction of Mn(IV) to Mn(III)/Mn(II). Notably, the HIS@Zn||MnO_2_ full cell delivers higher peak currents, indicating enhanced reaction kinetics.

Rate capability and cycling performance measurements further demonstrate the pronounced advantages of HIS@Zn. At current densities of 0.2, 0.5, 1, 2, and 5 A g^−1^ (Figure 6b), the HIS@Zn||MnO_2_ full cell achieves discharge capacities of 233.4, 180.44, 137.6, 104.4, and 72.2 mAh g^−1^, respectively, which are markedly superior to those of the Zn||MnO_2_ counterpart (140.2, 77.6, 50.1, 35.8, and 20.7 mAh g^−1^). Moreover, when the current density is restored from 5 A g^−1^ to 0.2 A g^−1^, the capacity of the HIS@Zn||MnO_2_ full cell is almost fully recovered, indicating excellent structural and electrochemical robustness. At a current density of 1 A g^−1^, the Zn||MnO_2_ full cell delivers an initial capacity of 160.5 mAh g^−1^ but suffers from rapid capacity fading upon cycling (Figure S31). In contrast, the HIS@Zn||MnO_2_ full cell maintains high discharge capacities over different cycles and exhibits more stable voltage plateaus (Figure 6c). Even after 700 cycles, the capacity retention of the HIS@Zn||MnO_2_ full cell remains as high as 92.33%, which is far superior to that of the Zn||MnO_2_ full cell (34.91%) (Figure 6d). The self‐discharge behavior of the full cells was also examined. As shown in Figure 6e,f, after resting for 24 h, the Zn|| MnO_2_ full cell retains only 80.81% of its capacity, whereas the HIS@Zn|| MnO_2_ full cell maintains a high capacity retention of 97.38%, demonstrating that the HIS@Zn anode effectively suppresses self‐discharge in the full cells.

The stability of the HIS@Zn electrode under full‐cell operating conditions was further investigated. As revealed in Figure S32 and Figure 6g, bare Zn suffers from dendrite penetration through the separator during cycling, accompanied by the formation of ZSH by‐products and a low I_(002)_/I_(101)_ ratio of only 0.39. In sharp contrast, the HIS@Zn electrode remains dense and intact, exhibits an exceptionally high I_(002)_/I_(101)_ ratio of 10.88, and shows no obvious diffraction signals associated with by‐products. These results collectively demonstrate that HIS@Zn maintains excellent structural and interfacial stability in full cell, thereby significantly enhancing both the rate capability and cycling performance of aqueous Zn‐MnO_2_ batteries.

## 3 Conclusions

In summary, an ultrathin Zn(OH)_2_‐HIS hybrid SEI was successfully constructed on the zinc anode surface by employing histidine (HIS), which features multiple pH‐buffering groups and abundant hydrogen‐bond donors/accepters, to address the interfacial challenges of aqueous zinc‐ion batteries. Our results demonstrate that this SEI stabilizes the interfacial pH via a synergistic mechanism of chemical buffering and physical blocking. Specifically, the imidazole and amino groups of the SEI dynamically buffer the electrode/electrolyte interfacial pH through reversible protonation /deprotonation processes. Simultaneously, the hydrogen‐bond donors/acceptors provided by the SEI efficiently disrupt the hydrogen‐bond network of water and reduce the coordination number of solvated Zn^2+^, thus mitigating water‐induced parasitic reactions. Furthermore, the HIS@Zn electrode effectively regulates the interfacial Zn^2+^ plating/stripping behavior and induces preferential Zn(002) crystallographic orientation during deposition and dissolution, even during long‐term cycling. Benefiting from these synergistic effects, the HIS@Zn electrode exhibits outstanding electrochemical performance. Zn||Zn symmetric cells operate stably for up to 1350 h at 10 mA cm^−2^ and 5 mAh cm^−2^, maintain over 300 h of cyclic life at an ultrahigh depth of discharge of 81%, and achieve a high Coulombic efficiency of 99.44%. Moreover, the HIS@Zn||MnO_2_ full cell delivers an initial discharge capacity of 146.6 mAh g^−1^ at 1 A g^−1^ and retains 92.33% of its capacity after 700 cycles. This study provides a novel interface design strategy for the development of high‐performance and long‐lifespan aqueous zinc‐ion batteries by constructing an SEI endowed with pH buffering, interfacial hydrogen bond network regulation, and Zn (002) oriented deposition/stripping.

## Author Contributions

**Kun Luo**: Writing – original draft, Writing – review and editing, supervision. **Zhihong Luo**: writing – review and editing, writing – original draft, resources, supervision, formal analysis, project administration. **Aijing Ma**: writing – original draft, writing – review and editing. **Jianwei Lu**: conceptualization, investigation, methodology, software, writing – review and editing, writing – original draft. **Weiwei Lei**: funding acquisition, writing – review and editing, writing – original draft. **Yimin Chen**: conceptualization, investigation, writing – original draft, writing – review and editing, software, methodology. **Dan Liu**: writing – original draft, writing – review and editing. **Anjun Hu**: writing – original draft, writing – review and editing, methodology, investigation. **Xiyuan Zhong**: methodology. **Qi Liu**: conceptualization, investigation, methodology, software, writing – review and editing, writing – original draft. **Yibing Li**: writing – original draft, writing – review and editing. **Xiangqun Zhuge**: conceptualization. **Huaichong Sun**: investigation.

## Conflicts of Interest

The authors declare no conflicts of interest.

## Supporting information




**Supporting File**: advs76765‐sup‐0001‐SuppMat.docx.

## Data Availability

The data that support the findings of this study are available from the corresponding author upon reasonable request.
