# Supplementary material for: Dynamic Interfacial pH Stabilization and (002) Oriented Deposition Enabled by Histidine‐Induced Solid Electrolyte Interphase for Highly Reversible Zn Anodes
Source: Adv Sci (Weinh). 2026 Jul 31:e76765. Online ahead of print. doi: 10.1002/advs.76765 (PMC13426870; doi:10.1002/advs.76765)
Supplement: Supplementary file 1 — Supporting File: advs76765‐sup‐0001‐SuppMat.docx. [file ADVS-9999-e76765-s001.docx]

Supporting Information

**Dynamic Interfacial pH Stabilization and (002) Oriented Deposition Enabled by Histidine-Induced Solid Electrolyte Interphases for Highly Reversible Zn Anodes**

*Qi Liu^1#^, Yimin Chen^3#^, Jianwei Lu^2#^, Xiangqun Zhuge^3^, Xiyuan Zhong^1^, Huaichong Sun^1^, Yibing Li^1^, Zhihong Luo^1*^, Kun Luo^3*^, Weiwei Lei^2*^, Dan Liu^2^, Anjun Hu^5*^, Aijing Ma^4*^*

**Experimental Section**

**Preparation of Bare Zn and HIS@Zn anodes**

Commercial Zn foil was cut into Zn sheets (0.05 mm thick, 16 mm diameter and weighing 0.06 g to prepare bare Zn anodes. A total of 0.078 g of histidine (HIS) was dissolved in 50 mL of deionized water to prepare a 10 mmol L^-1^ solution. The cut zinc anodes were immersed in the HIS solution for 3, 5, 7 h, rinsed with deionized water, and then dried in an oven at 60 ℃ for 10 h to obtain the HIS@Zn-3h, HIS@Zn, HIS@Zn-7h anodes.

**Preparation of MnO_2_ cathode**

A 0.15 mol L^-1^ MnSO_4_ solution was added dropwise to a 0.10 mol L^-1^ KMnO_4_ solution with stirring for 30 minutes. The mixture was then quickly transferred to a hydrothermal autoclave and placed in an oven for hydrothermal reaction at 160℃ for 12 h. After the reaction, the product was separated by centrifugation, washed three times with deionized water, and finally dried in an oven at 60℃ to obtain MnO_2_ powder. The MnO_2_ was mixed with carbon black and PVDF in a weight ratio of 7:2:1. The as-prepared ink was coated on a stainless-steel mesh and dried at 60℃ for 12 h to obtain a MnO_2_ cathode with an areal loading mass of about 2.0 mg cm^-2^.

**Battery assembling and testing**

Bare Zn, HIS@Zn, Cu foil with a diameter of 16 mm, and MnO_2_ cathode (loading of 2.0 mg cm^-2^) with a diameter of 14 mm were used as electrodes. 2 M ZnSO_4_ was used as the electrolyte, and glass fibre with a diameter of 18 mm was used as the separator. All batteries were assembled with CR2032 coin cells, and Zn||Zn symmetric cells were assembled using bare Zn or HIS@Zn as the electrode. Zn||Cu half-cells were constructed with bare Zn as the negative electrode and HIS@Zn as the positive electrode. Zn||MnO_2_ full cells were built with bare Zn as the anode and HIS@Zn as the cathode. The battery performance was recorded on a standard battery testing system (CT-3008W-5V10mA, Neware Technology Limited). The plating/stripping process was measured by galvanostatic charge/discharge with Zn||Zn symmetric cell at the current density of 10 mA cm^-2^ for 2 h, the cyclic performance was tested at 10, 16, 20 mA cm^-2^ with the capacity of 5, 16, 20mAh cm^-2^. Coulombic Efficiency was tested with Zn||Cu half-cell, where Zn^2+^ was deposited onto Cu electrode at a current density of 0.5 mA cm^-2^ for 4 h and then cycled at a current density of 0.5 mA cm^-2^ and a capacity of 0.5 mAh cm^-2^. The cyclic and rate performance of Zn||MnO_2_ full cells were tested at the current densities of 0.2, 0.3, 0.5, 1, 2, and 5 A g^-1^ in the potential range of 0.8 V to 1.8 V.

**Electrochemical measurement testing**

CV, EIS, I-t, Tafel, LSV, nucleation overpotential and CA curve tests were performed in an electrochemical workstation (Squidstat Plus, 1.1). CV curves for a Zn||SS cell were obtained in a voltage window of -0.2 V to 0.4 V at a scan rate of 20 mV s^-1^; CV curves for a Zn||MnO_2_ cell were obtained in the voltage range of 0.8 V to 1.8 V at scan rates ranging from 0.1 mV s^-1^. The LSV test was performed in a voltage range of -2.1 V to -1.0 V (vs. Ag/AgCl) at a scan rate of 5 mV s^-1^. EIS, I-t, Tafel, nucleation overpotential and CA curves were obtained from a Zn||Zn cell. The EIS measurement was performed over a frequency range of 10 mHz to 100 kHz. The CA measurement was performed under a constant overpotential of -150 mV.

The electroactive surface area of the Zn anode is estimated from the specific surface area of the peak current in the CV curve. The formula for estimating the *A_e_* using *i_p_* is as follows:^[1]^

$$i_{p}=3.67\times{10}^{5}n^{\frac{3}{2}}A_{e}cD^{\frac{1}{2}}\nu^{\frac{1}{2}}$$

where *i_p_* (mA) is the peak current, *n* (2) is the number of electrons, *A_e_* (cm^2^) is the electroactive surface area, *c* (2×10^-3^ mol cm^-3^) is the bulk concentration of Zn^2+^, *D* (cm^2^ s^-1^) is the diffusion coefficient of Zn^2+^, and *ν* (0.02 V s^-1^) is the scan rate.

**Material characterizations**

The scanning electron microscope (SEM, S4800, Hitachi) was employed to analyze the microcosmic surface morphology and related energy dispersive spectroscopy (EDS). High-resolution transmission electron microscopy images (HRTEM), elemental mapping, and selected-area electron diffraction (SAED) patterns were collected using a transmission electron microscope (TEM, JEM-2100F, JEOL). Fourier transform infrared (FTIR) and Raman spectrometer (Thermo Nicolet 6700-NXR, Thermo Scientific) were used to characterize the structures. The contact angle (JGW-360A) between the anode and electrolyte was used to characterise electrolyte wettability. X-ray diffractometer (XRD, X’ Pert Pro, PANalytical B.V.) with a Cu-Kα target under a voltage of 40 KV was enlisted to obtain information on the crystal structure of the anode. The optical observation of the deposition process was conducted on a dendrite observation microscope system (YUESCOPE YM710TR-Z). *In-situ* Raman spectra were tested on a Raman spectrometer (Thermofisher, DXR2) with a home-made cell at a current density of 0.1 mA cm^-2^ and a capacity of 0.1 mAh cm^-2^, and data were recorded every 4 mins for 1 min each.

**Computational methods**

All calculations were performed by Materials Studio 2020. Generalized gradient approximation (GGA) parameterized by Perdew-Burke-Ernzerhof (PBE) formula was employed for evaluating the electron exchange correlation energy. The DFT-D3 method of Grimme was used to describe the weak dispersion forces. The structural parameters and all the atoms were fully optimized until the Hellman-Feynman forces were less than 0.002 eV Å^-1^. Energy and force will not reach convergence until lower than 1×10^-5^ eV and 0.02 eV Å^-1^, respectively. Then, the migration barrier was obtained by the TS search. The electrostatic potential (ESP) and dipole moment were obtained from optimized molecules. Subsequently, A plane-wave basis set with a kinetic energy cutoff of 550 eV was employed. Brillouin-zone integrations were performed using a Monkhors-Pack k-point mesh with a reciprocal-space resolution of 0.04 Å^-1^. All surfaces contain 3 layers of zinc atoms, 1 layer of zinc hydroxide molecules, and the HIS was absorbed on each surface. A vacuum layer of 15 Å was introduced along the surface normal direction to avoid spurious interactions between periodically repeated slabs. Structural optimizations were considered converged when the total energy change was less than 2.0×10^-5^ eV per atom, the maximum force on each atom was below 0.01 eV Å^-1^, the maximum stress was smaller than 0.02 GPa, and the maximum atomic displacement was less than 5.0×10^-4^ Å, corresponding to the default convergence thresholds in CASTEP. All atomic positions were fully relaxed during the optimization.

The binding energy between different sites of histidine and zinc hydroxide was computed as follows:^[2]^

$$E_{\text{bind}}=E_{\text{complex}}-E_{\text{His}}-E_{\text{Zn}\left( \text{OH} \right)_{\text{2}}}$$

where $E_{\text{complex}}$, $E_{\text{His}}$ and $E_{\text{Zn}\left( \text{OH} \right)_{\text{2}}}$ stand for the total energy of the complex after histidine binds to zinc hydroxide at a specific site, the monomer energy, and the energy of zinc hydroxide.

The adsorption energy between the hydrogen ion and different sites was defined as the following equation: ^[3]^

$$E_{\left( ad \right)}=E_{\left( *adsorbent \right)}-E_{\left( * \right)}-E_{\left( adsorbent \right)}$$

where$E_{\left( *adsorbent \right)}$, $E_{\left( * \right)}$and $E_{\left( adsorbent \right)}$ represent the total energy of *adsorbent, * and adsorbent molecule, respectively.

The deposition energy of Zn^2+^ ion was calculated as follows:^[3]^

$$E_{dp}=E_{S+Zn}-E_{S}-E_{Zn^{2+}}$$

where the $E_{S+Zn}$ represents the energy of the surface absorb a Zn atom, the $E_{S}$ represents the energy of clean surface, and the $E_{Zn^{2+}}$ represents the energy of Znic-ion.

The dissolution energy barrier of the Zn atom was calculated as follows:^[3]^

$$E_{ds}=E_{S-Zn}+E_{Zn^{2+}}-E_{S}$$

where the $E_{S-Zn}$ represents the energy of the surface absent a Zn atom, the $E_{Zn^{2+}}$ represents the energy of Znic-ion, and the $E_{S}$ represents the energy of clean surface.

**COMSOL Simulation**

Finite element simulations were performed using COMSOL Multiphysics 6.2. The current density and ion concentration fields were modelled utilizing the “Tertiary Current Distribution” physics interface. The simulation used the Butler-Volmer expression to describe the reaction kinetics of all electrodes, while ion migration followed the Nernst-Planck formulation. The surface morphologies of bare Zn and HIS@Zn electrodes were constructed based on SEM observations. Both models adopted a simplified geometric domain, where the electrode length was set to 10 μm and the inter-electrode spacing to 7 μm. The simulations primarily focused on the electrode/electrolyte interface. Boundary conditions were applied by setting the positive electrode at zero electric potential, while the negative electrode was assigned a potential corresponding to the cell polarization voltage. The system temperature was maintained at 298 K, and the initial ion flux for the ZnSO_4_ electrolyte was set to 2 mol L^-1^.

**Supplementary Figures and Tables**

**
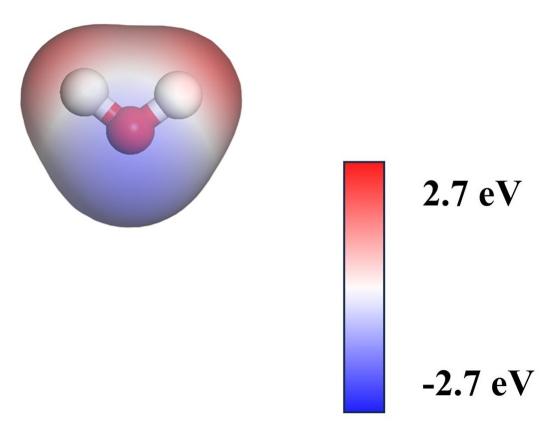
**

**Figure S1.** ESP of the H_2_O molecule.


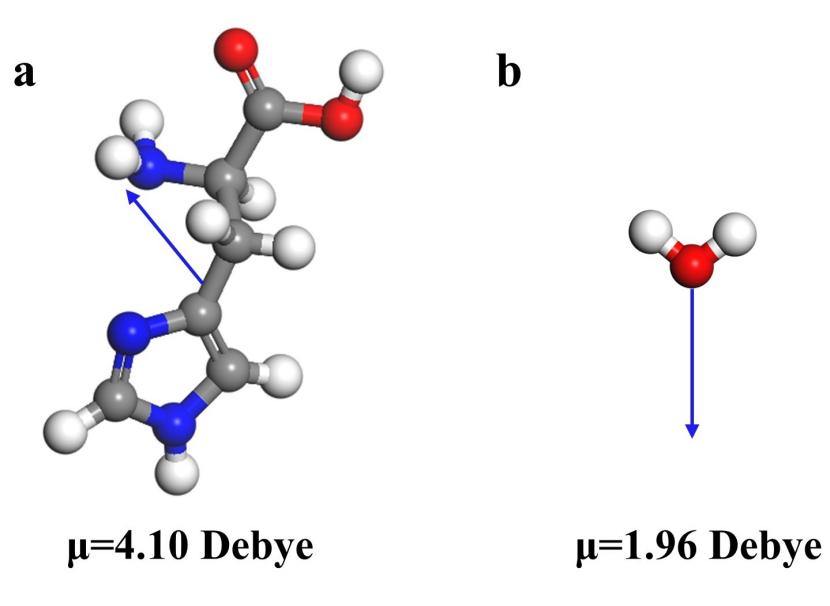


**Figure S2.** Calculated dipole moments of (a) HIS molecule and (b) H_2_O molecule.


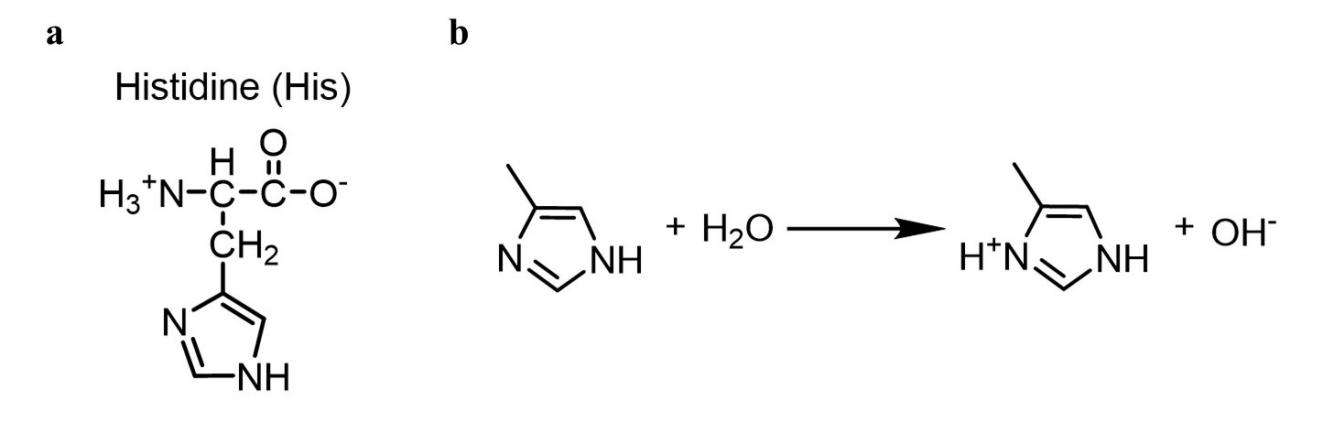


**Figure S3.** (a) The molecular formulas of HIS and (b) the hydrolysis of its imidazole ring side chain.


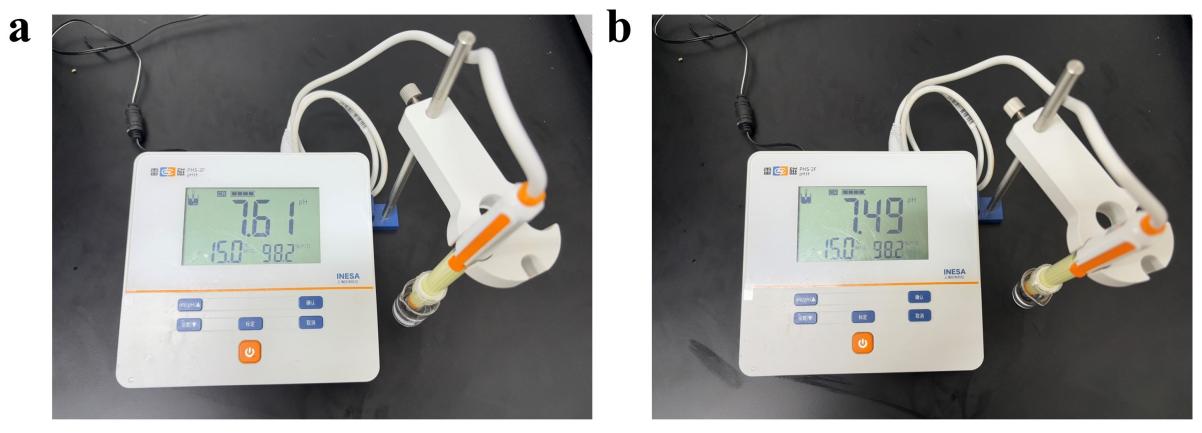


**Figure S4.** The pH value of the HIS solution (a) before and (b) after zinc sheets immersion.


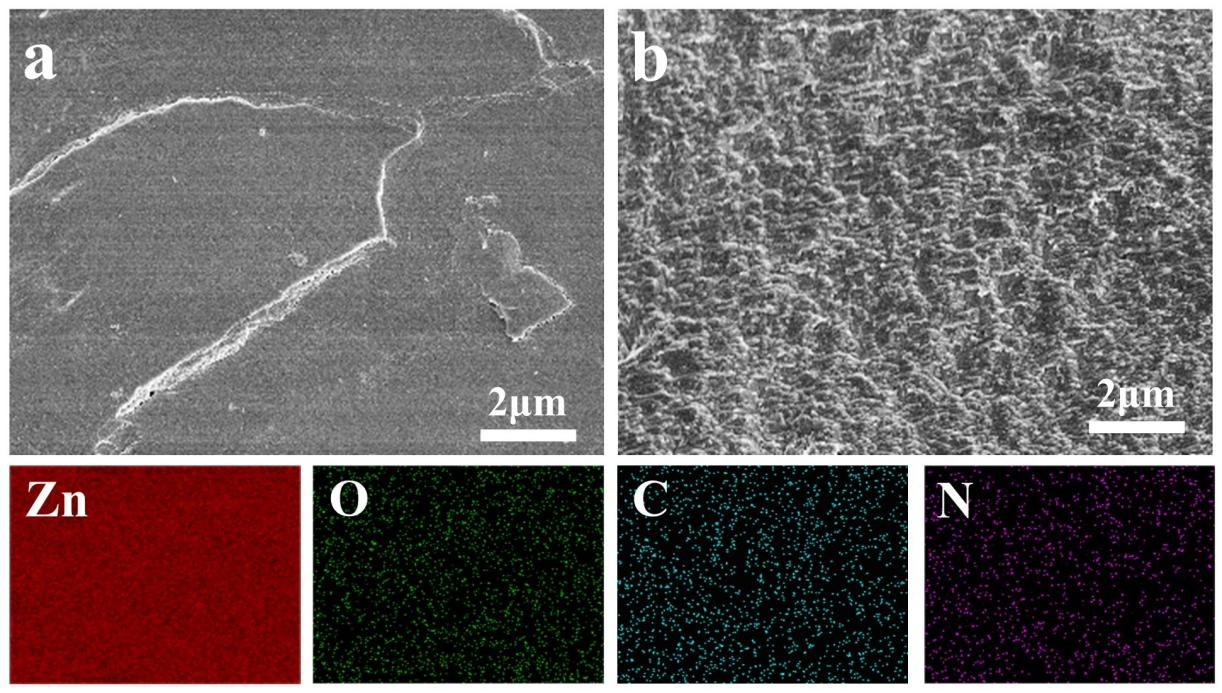


**Figure S5.** SEM images of (a) bare Zn, (b) HIS@Zn and corresponding elemental distribution of HIS@Zn.


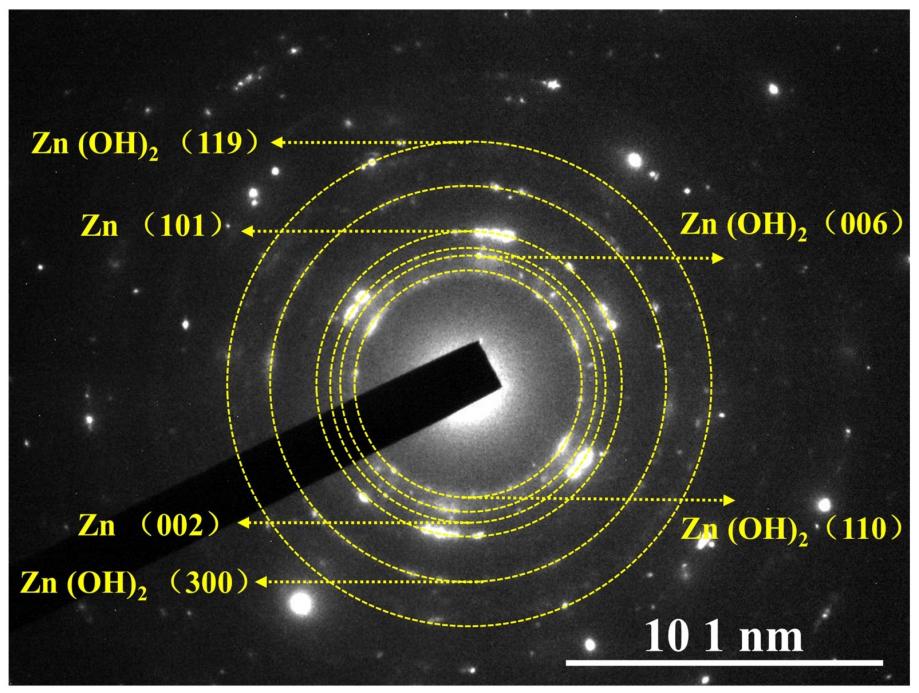


**Figure S6.** SAED pattern of the material scraped from the HIS@Zn electrode.


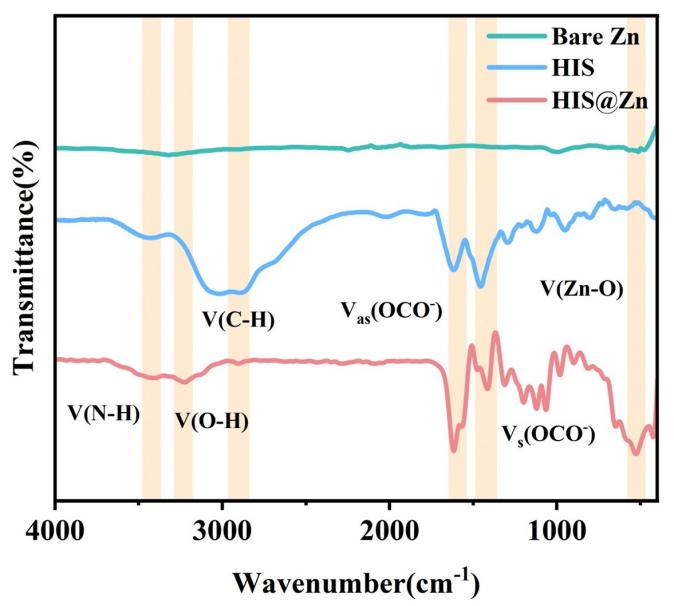


**Figure S7.** FTIR spectra of bare Zn, HIS and HIS@Zn.


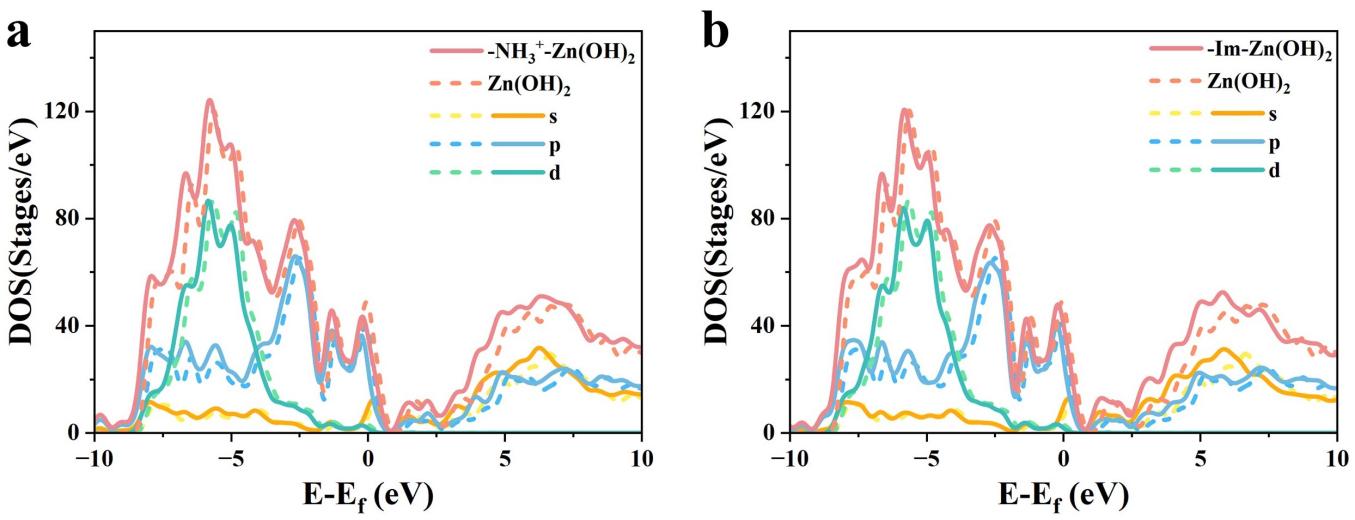


**Figure S8.** Comparison of density of states (DOS) between Zn(OH)_2_ and (a) -NH_3_^+^-Zn(OH)_2_ system or (b) -Im-Zn(OH)_2_ system.


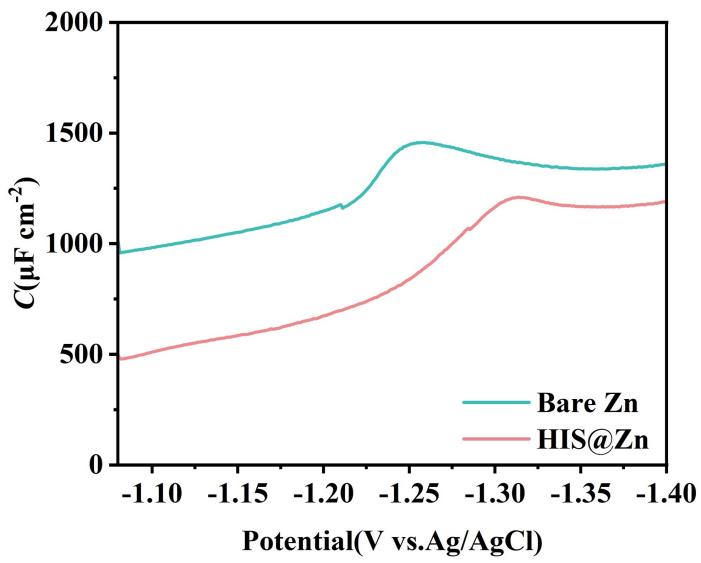


**Figure S9.** Differential capacitance curves of bare Zn and HIS@Zn.


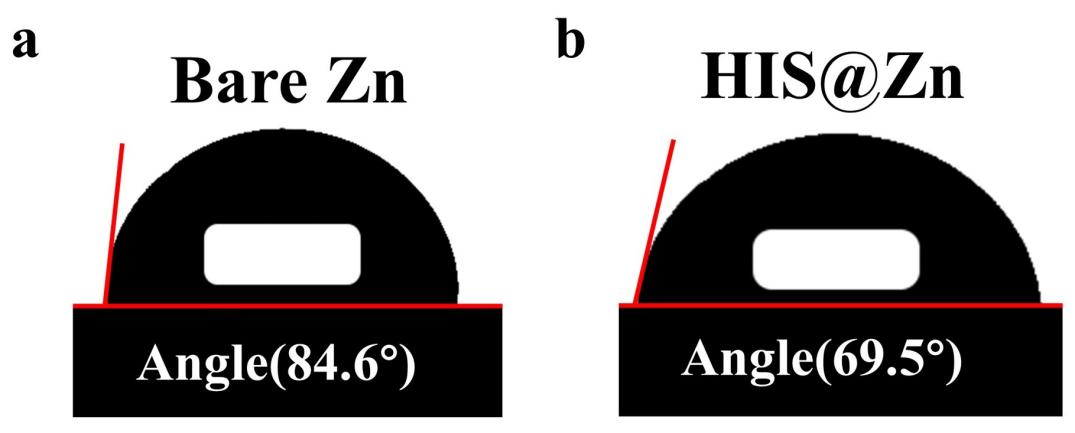


**Figure S10.** Contact angle of electrolyte on (a) bare Zn and (b) HIS@Zn.


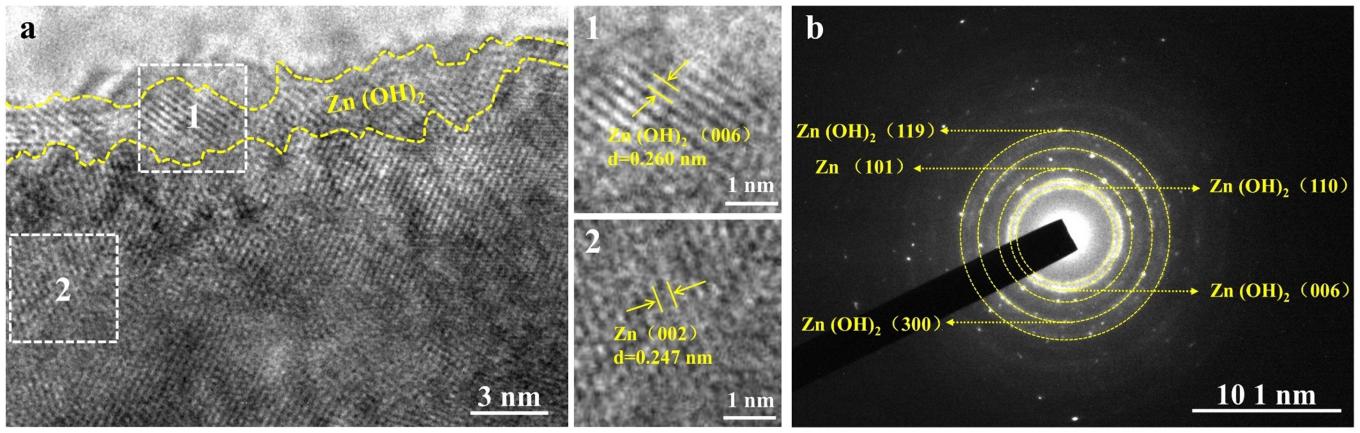


**Figure S11.** (a) HRTEM and (b) SAED of the material scraped from the HIS@Zn after 24 h immersion.


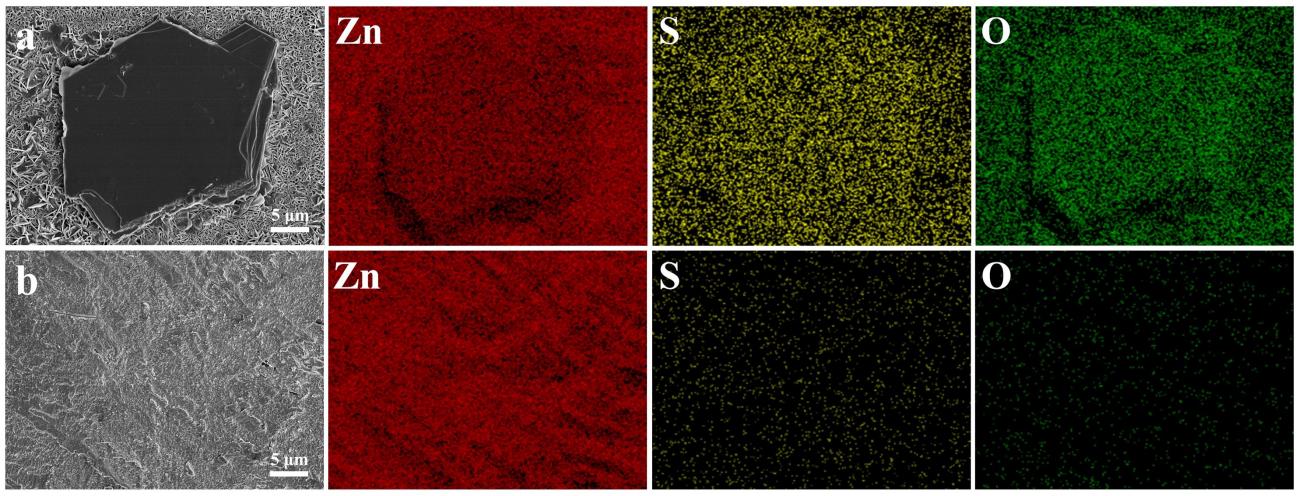


**Figure S12.** SEM images of (a) bare Zn and (b) HIS@Zn after immersing for 10 days with the corresponding elemental distribution of Zn, S, and O.


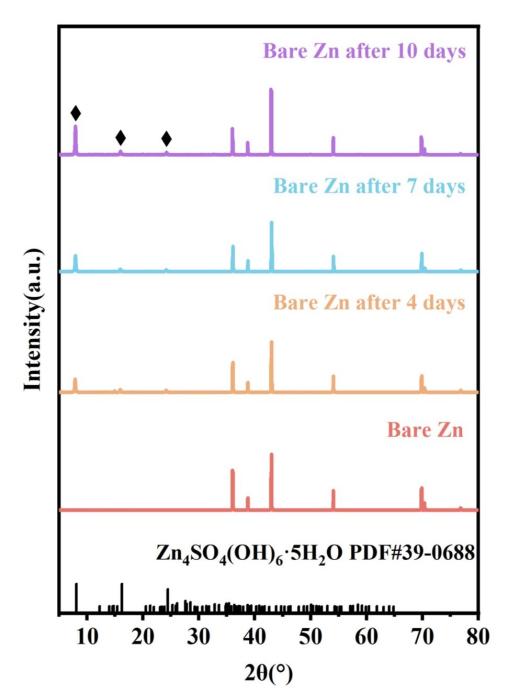


**Figure S13.** XRD patterns of bare Zn after immersion for different days.


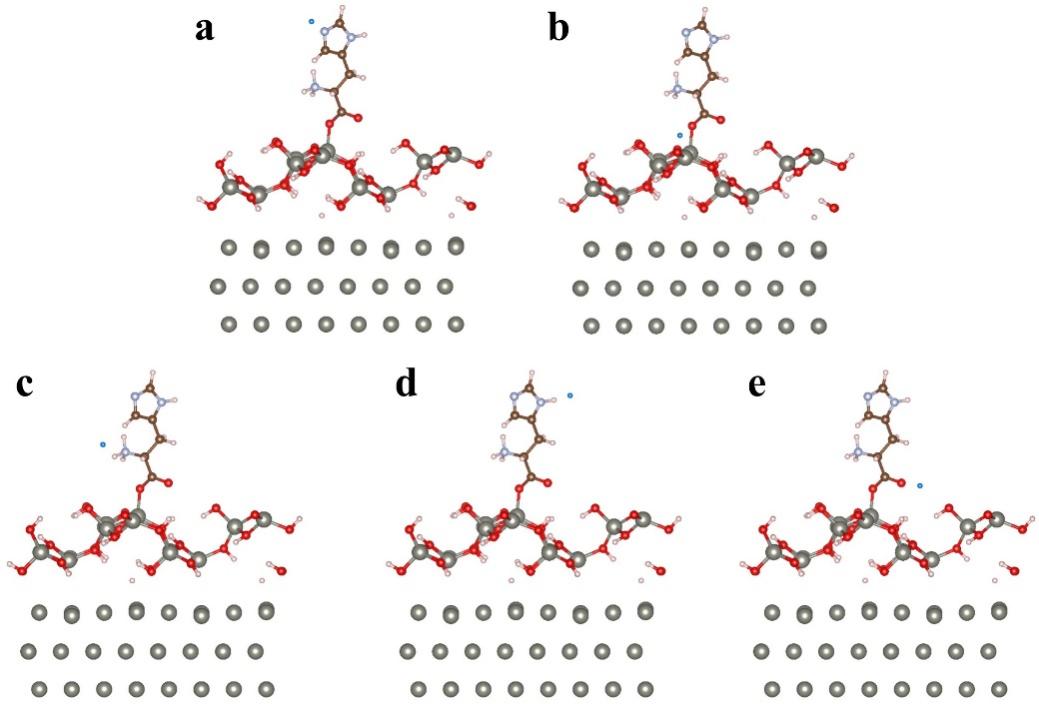


**Figure S14.** Computational models for the adsorption energy of H^+^at (a) the N^τ^ site, (b) the Zn(OH)_2_ site, (c) the NH_3_^+^ site, (d) the N^π^ site and (e) the COO^−^ site.


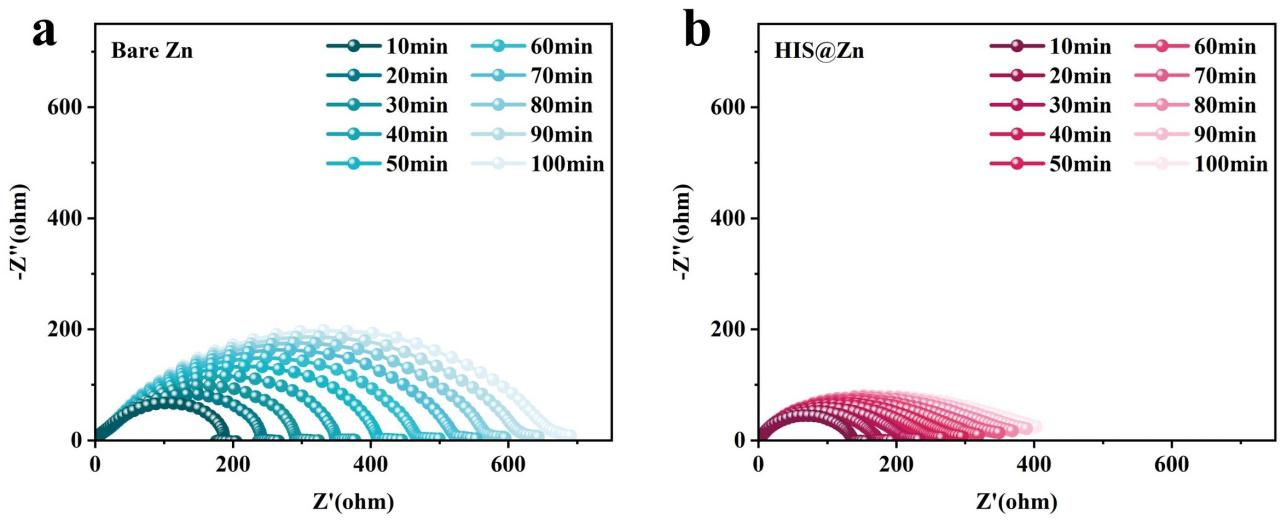


**Figure S15.** The EIS plots of Zn||Zn cell with (a) bare Zn and (b) HIS@Zn during continuous rest.


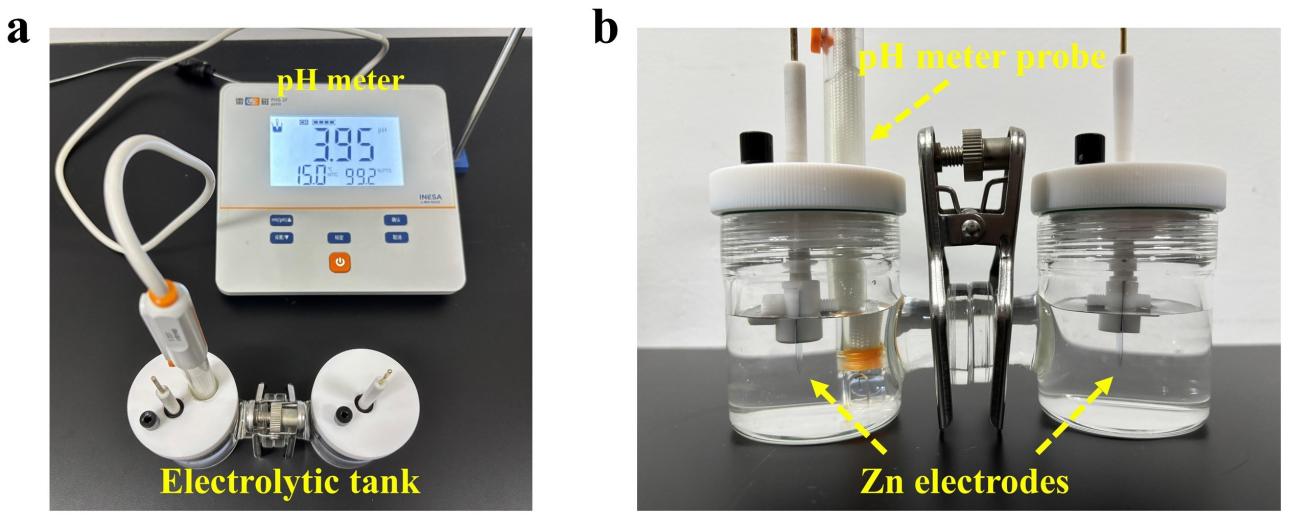


**Figure S16.** (a) Digital photographs of the home-made in situ pH measurement setup and (b) the magnified view of the assembled Zn||Zn cell in the electrolytic tank.


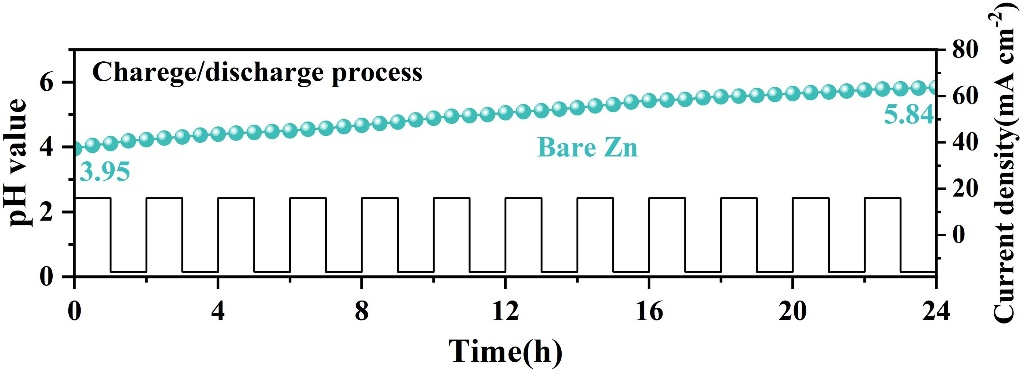


**Figure S17.** In situ pH test of Zn||Zn cells with bare Zn during the charge/discharge process at the current density of 16 mA cm^-2^ and capacity of 16 mAh cm^-2^.


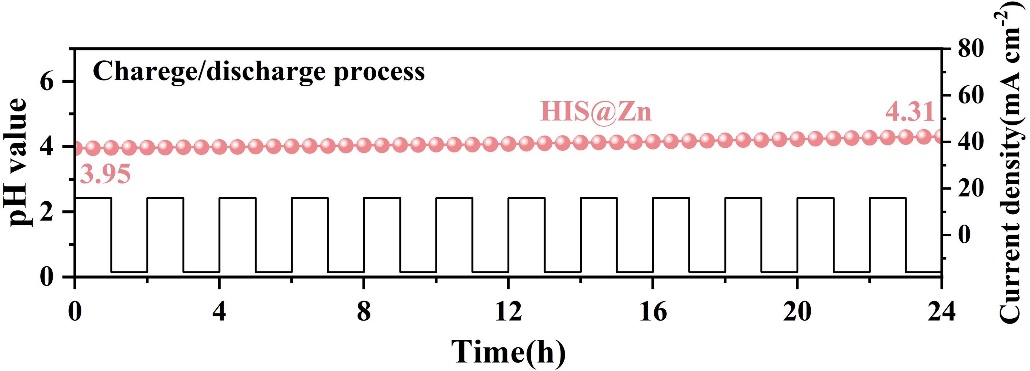


**Figure S18.** In situ pH test of Zn||Zn cells with HIS@Zn during the charge/discharge process at the current density of 16 mA cm^-2^ and capacity of 16 mAh cm^-2^.

**
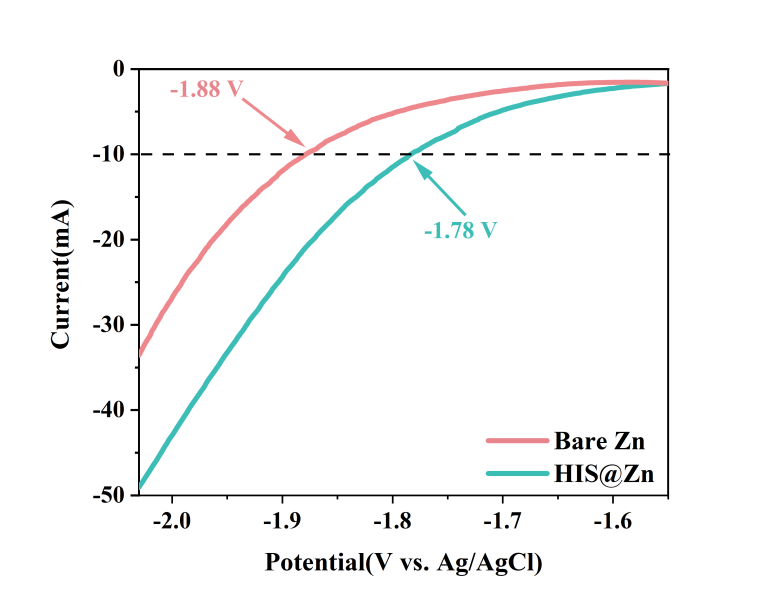
**

**Figure S19.** LSV curves of bare Zn and HIS@Zn.


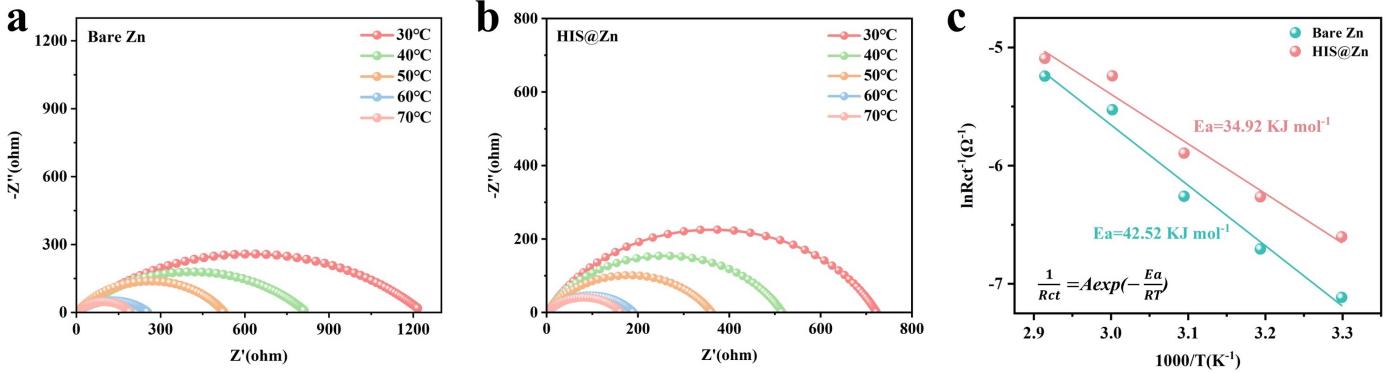


**Figure S20.** The EIS plots at different temperature of Zn||Zn cell with (a) bare Zn, (b) HIS@Zn and (c) comparison of Arrhenius activation energies.

The EIS of Zn||Zn cell at different temperatures was measured. The linear relationship between charge transfer resistance (*R_ct_*) and temperature can be obtained by fitting the Arrhenius equation:^[2]^

$$\frac{1}{R_{ct}}=A\exp\left( \frac{-E_{a}}{RT} \right)$$

Where *R_ct_* (Ω) is the charge transfer resistance, *A* is the constants, *T* (K) is the Kelvin temperature, *R* (J K^-1^ mol^-1^) is the ideal gas constant and *E_a_* is the activation energy, respectively.


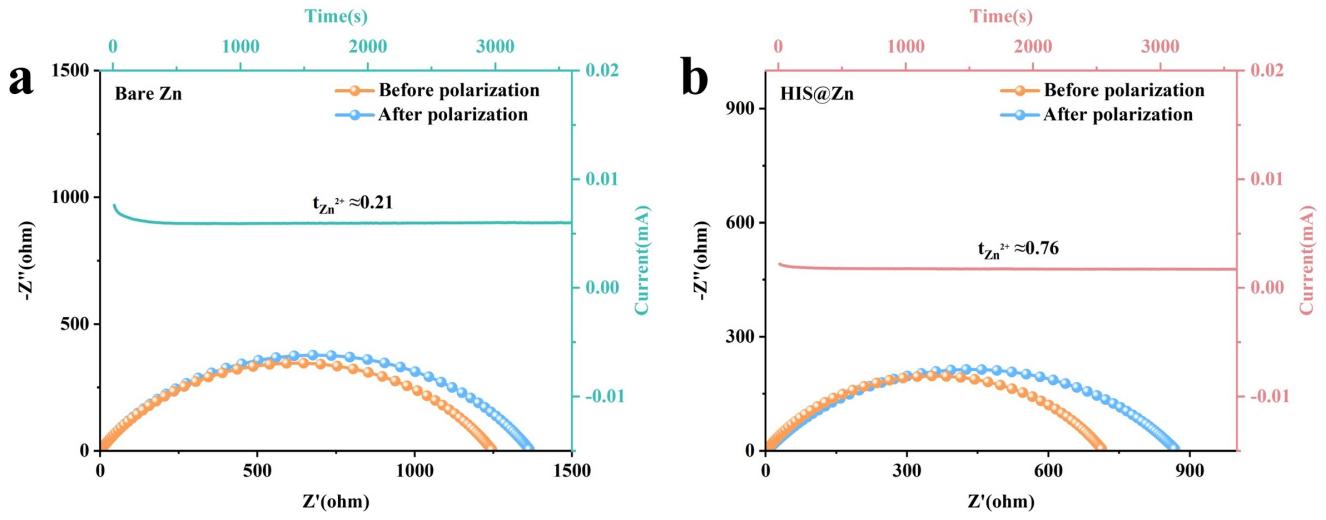


**Figure S21.** I-t curves at the bias potential of 10 mV and EIS plots before and after polarization of Zn||Zn cell with (a) bare Zn and (b) HIS@Zn.

Zn||Zn cell with bare Zn and HIS@Zn was applied to measure Zn^2+^ transference number by using Bruce-Vincent method, the I-t curves were recorded with a bias voltage of 10 mV, the EIS plots were tested before and after polarization. The Zn^2+^ transference number is calculated by the following formula:^[2]^

$$t_{\text{Z}\text{n}^{\text{2+}}}=\frac{I_{ss}\left( V-I_{0}R_{0} \right)}{I_{0}\left( V-I_{ss}R_{ss} \right)}$$

where *V* (10 mV) is the applied bias voltage, *I_ss_* (mA) and *R_ss_* (Ω) are the steady state current and resistance, respectively, and *I_0_* (mA) and *R_0_* (Ω) are the initial current and resistance.


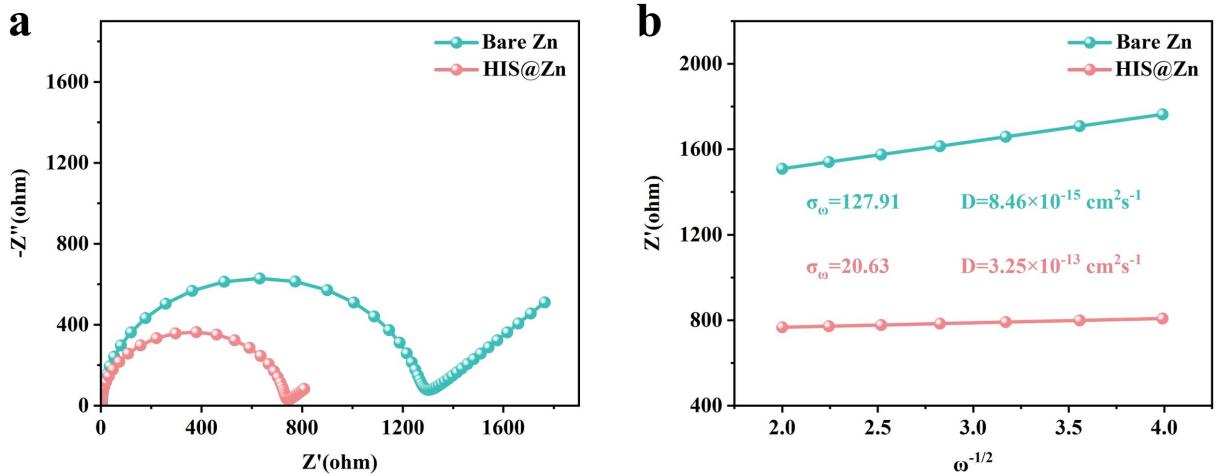


**Figure S22.** The EIS plots of Zn||Zn cell with (a) bare Zn, HIS@Zn and (b) the fitting curves of Z' versus ω^−1/2^ in the low-frequency region.

The EIS of Zn||Zn cell was measured to obtain the the Zn^2+^ diffusion coefficient and the Warburg coefficient. By extracting the linear slope between the real part of impedance in the low-frequency Warburg region and the square root of the corresponding angular frequency, the Zn^2+^ diffusion coefficient can be calculated by the following formula:

$$D=0.5\left( \frac{RT}{An^{2}F^{2}\sigma_{\omega}C} \right)^{2}$$

where *D* (cm^2^ s^-1^) represents the ion diffusion coefficient, *R* (J K^-1^ mol^-1^) is the gas constant, *T* (K) is the Kelvin temperature, *A* (cm^2^) is the surface area of the electrode, *F* (C mol^-1^) is the Faraday constant, *C* (mol cm^-3^) is the molar concentration of zinc ions in the electrolyte, and *σ_ω_​* is the Warburg factor, which can be calculated according to the following formula:

$$\sigma_{\omega}=\frac{Z^{'}}{\omega^{-0.5}}$$

where *Z′* is the real part of impedance in the low-frequency Warburg region, and *ω* is the angular frequency in this region.


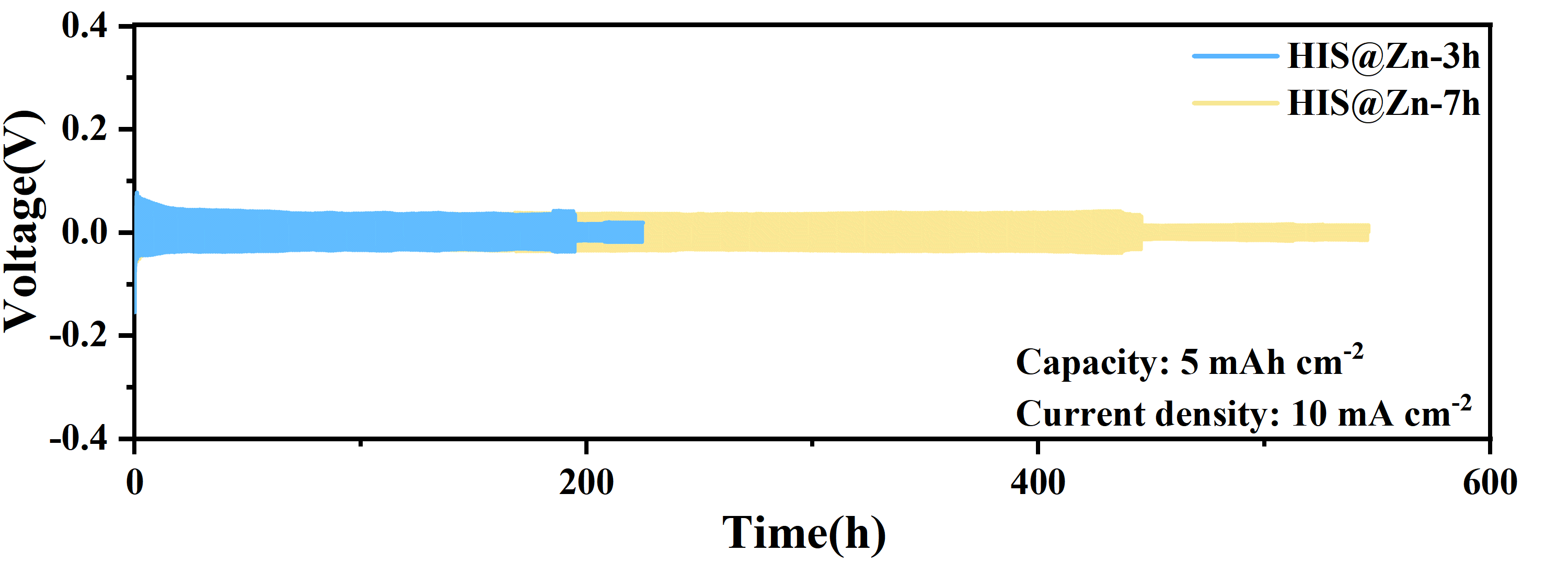


**Figure S23.** Cyclic performance of Zn||Zn cells with HIS@Zn-3h and HIS@Zn-7h at the current density of 10 mA cm^-2^ and capacity of 5 mAh cm^-2^.


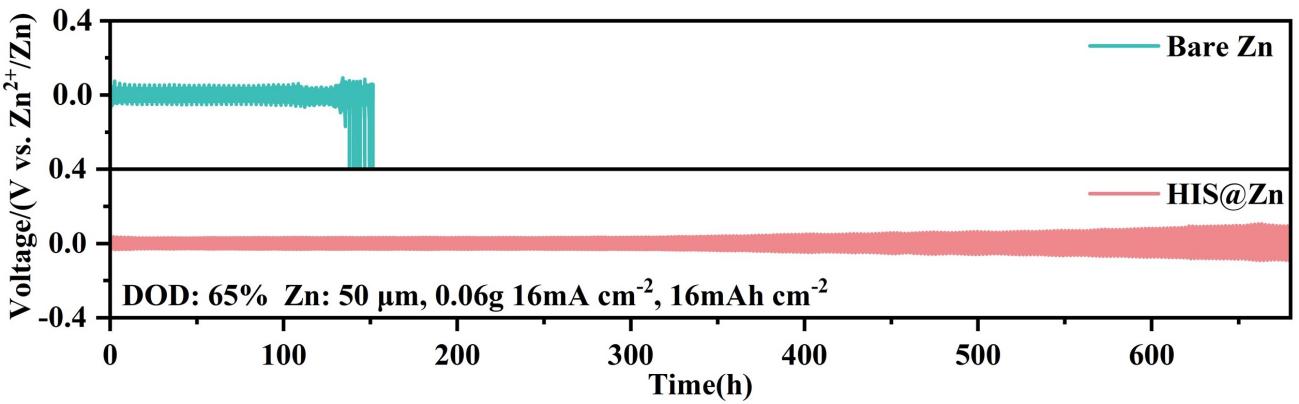


**Figure S24.** Cyclic performance of Zn||Zn cell with bare Zn and HIS@Zn at the current density of 16 mA cm^-2^ and capacity of 16 mAh cm^-2^ .

The DOD of Zn anode was calculated using the following equation:^[4]^

$$DOD=\frac{It}{mM}\times100\%$$

where *I* (mA) is the applied current density, *t* (h) is the discharge time, *m* (g) is the mass of the Zn anode, *M* (mAh g^-1^) is the theoretical specific capacity of Zn anode (820 mAh g^-1^).


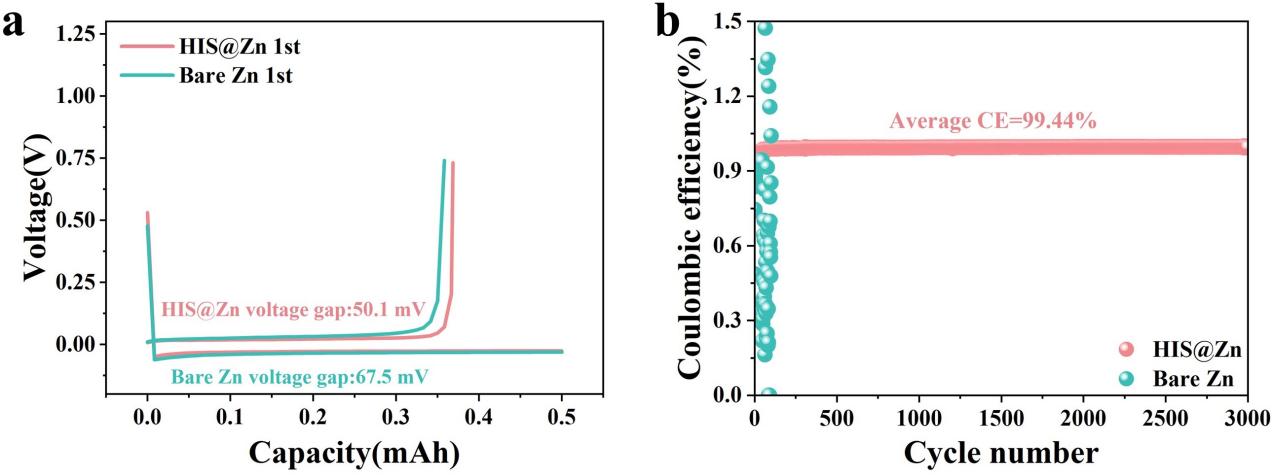


**Figure S25.** (a) Charge/discharge curves of Zn||Cu cell with bare Zn and HIS@Zn, and (b) the corresponding coulombic efficiency.


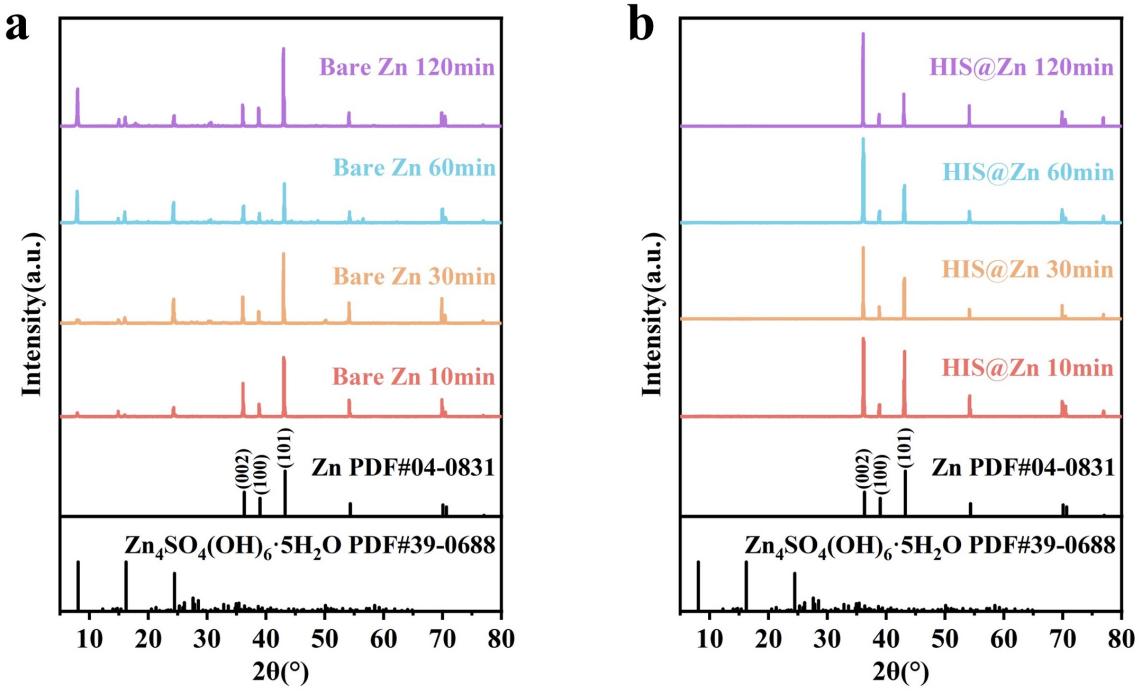


**Figure S26.** The XRD patterns of (a) bare Zn and (b) HIS@Zn during the plating process at the current density of 10 mA cm^-2^.


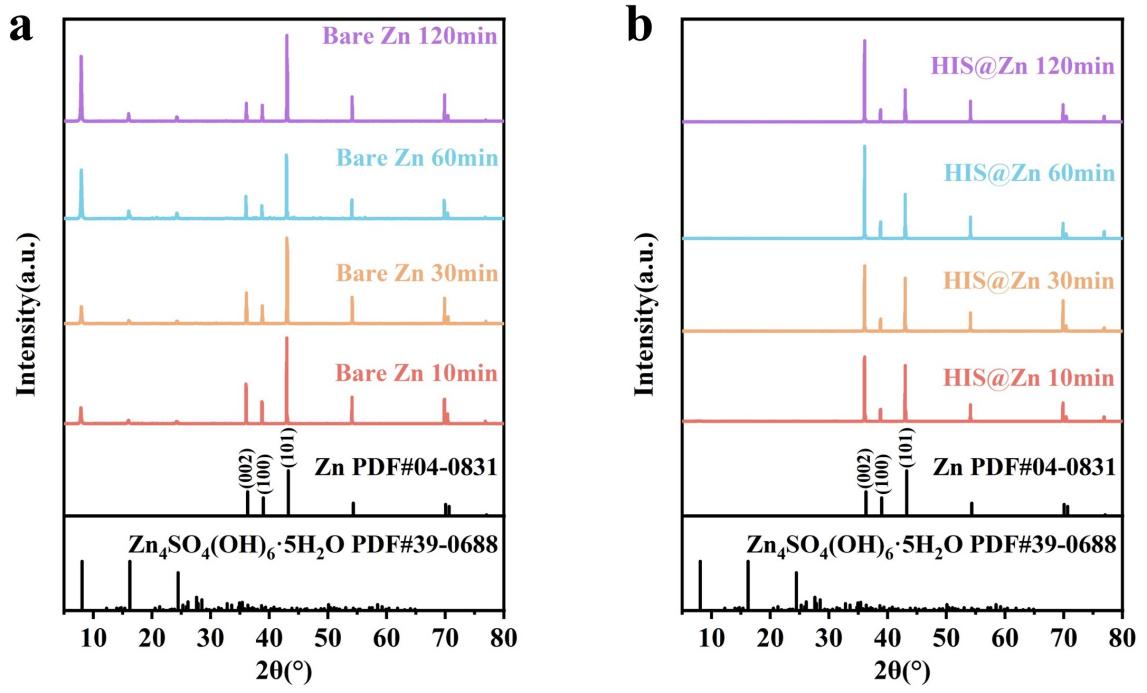


**Figure S27.** The XRD patterns of (a) bare Zn and (b) HIS@Zn during the stripping process at the current density of 10 mA cm^-2^.


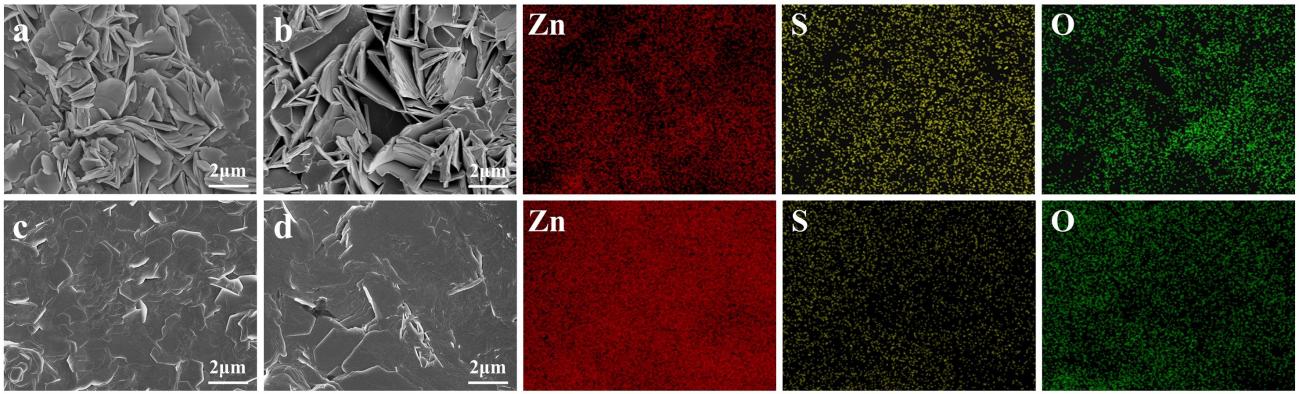


**Figure S28.** Surface morphology and Zn, S, O elemental distribution of (a、b) bare Zn anode after 10 and 100 cycles and (c, d) HIS@Zn anode after 10 and 1350 cycles at the current density of 10 mA cm^-2^ and capacity of 5 mAh cm^-2^.


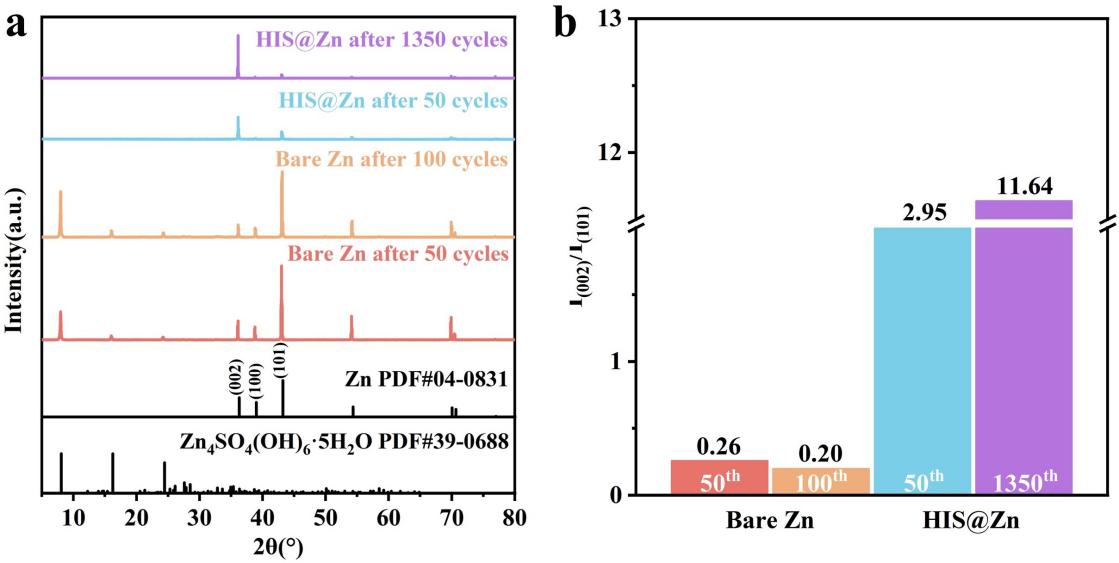


**Figure S29.** (a) The XRD patterns of bare Zn anode after 10 and 100 cycles and HIS@Zn anode after 10 and 1350 cycles. (b) The corresponding ratios of crystal planes.


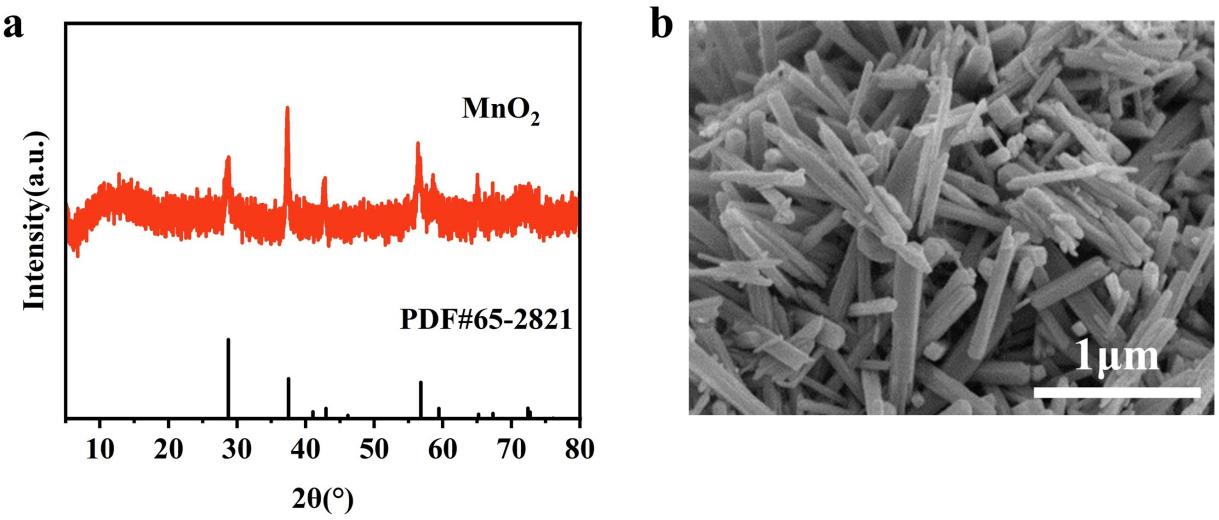


**Figure S30.** (a) XRD pattern and (b) SEM image of α-MnO_2_.


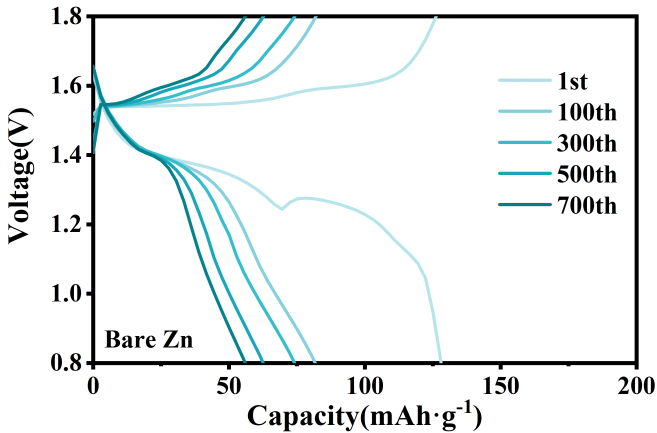


**Figure S31.** Galvanostatic charge-discharge profiles of Zn||MnO_2_ cell with bare Zn.


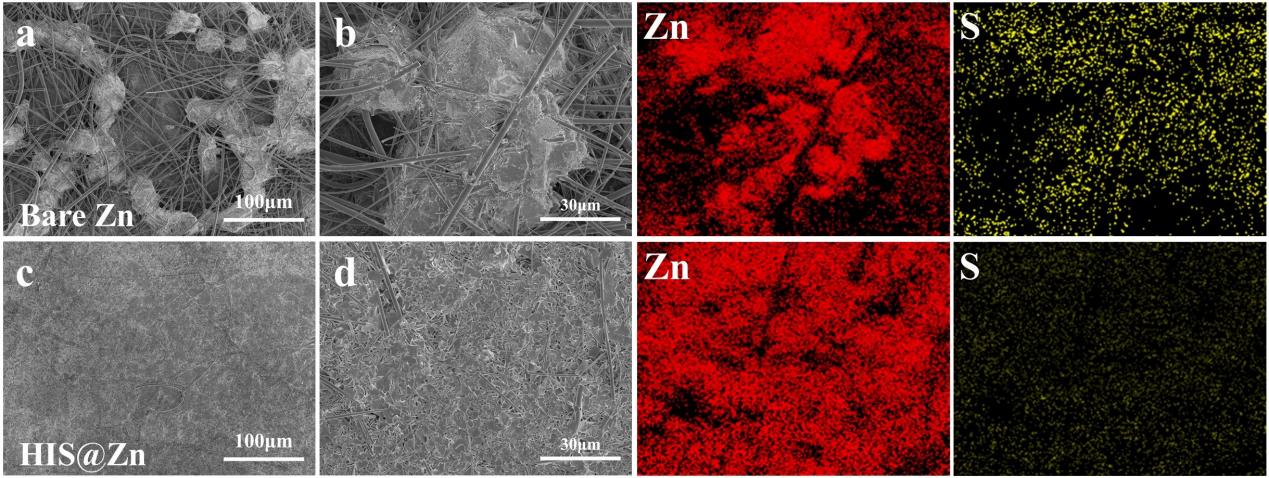


**Figure S32.** Surface morphology and Zn, S elemental distribution of (a、b) bare Zn anode and (c, d) HIS@Zn anode in Zn||MnO_2_ cell after 700 cycles at the current density of 1 A g^-1^.

**Table S1.** Comparison of cycling performance for this work with recently reported Zn||Zn symmetric cells in Figure 5h.

| Anode | Current density  /mA cm^-2^ | Areal capacity  /mAh cm^-2^ | Cyclic life/h | Reference |
| --- | --- | --- | --- | --- |
| HIS@Zn | 10 | 5 | 1350 | This work |
|  | 20 | 20 | 300 |  |
|  | 16 | 16 | 600 |  |
| Fs-Zn | 0.5 | 1.5 | 180 | [23] |
|  | 1 | 1 | 530 |  |
| CCu@Zn | 1 | 1 | 900 | [24] |
|  | 10 | 10 | 100 |  |
| NLC900@Zn | 2 | 1 | 1000 | [25] |
|  | 5 | 5 | 500 |  |
| PC@Zn | 0.5 | 0.5 | 1250 | [26] |
|  | 5 | 1 | 700 |  |
| LBL | 4 | 1 | 1200 | [27] |
|  | 8 | 4 | 600 |  |
| ZCSB@Zn | 10 | 2.5 | 1300 | [28] |
| Zn@H-SPEEK | 2 | 2 | 1230 | [29] |
| AIL-Zn | 1 | 1 | 1000 | [30] |
| ZnO-Zn | 10 | 1 | 1126 | [31] |
| Zn@COF/Nafion | 5 | 5 | 420 | [32] |
| PMMA@Zn | 10 | 5 | 1000 | [33] |
|  | 20 | 20 | 120 |  |
| PM@Zn | 20 | 1 | 200 | [34] |
| PFPE-Zn | 0.25 | 0.25 | 740 | [35] |

**References**

1. P. Wang, S. Liang, C. Chen, et al., “Spontaneous construction of nucleophilic carbonyl-containing interphase toward ultrastable zinc-metal anodes,” *Advanced Materials* 34 (2022): 2202733,

<https://doi.org/10.1002/adma.202202733>

2. A. Wu, S. Zhang, Q. Li, et al., “Multifunctional crown ether additive regulates desolvation process to achieve highly reversible zinc-metal batteries,” *Advanced Energy Materials* 15 (2025): 2404450,

<https://doi.org/10.1002/aenm.202404450>

3. M. Zhou, Z. Luo, J. Lu, et al., “Plane protection enabling (002) oriented plating and stripping processes for aqueous Zn-ion batteries,” *Energy & Environmental Materials* 8 (2025): e70056,

<https://doi.org/10.1002/eem2.70056>

4. Z. Luo, T. Xu, L. Cao, et al., “Caffeine-enhanced Zn (002) texture-oriented growth for stable aqueous zinc-ion batteries,” *Journal of Energy Chemistry* 107 (2025): 44-52,

<https://doi.org/10.1016/j.jechem.2025.02.066>
